# Slowing Down Zinc Electrodeposition Kinetics Can Maximize and Compromise Anode Stability: How Slow Is Too Slow?

**DOI:** 10.1002/anie.1181269

**Published:** 2026-03-10

**Authors:** Md. Arif Faisal, Taizhe Liu, Ashutosh Rana, James H. Nguyen, Saptarshi Paul, Ashutosh Bhadouria, Brian M. Tackett, Jeffrey E. Dick

**Affiliations:** ^1^ Department of Chemistry Purdue University West Lafayette Indiana USA; ^2^ Davidson School of Chemical Engineering Purdue University West Lafayette Indiana USA; ^3^ Elmore Family School of Electrical and Computer Engineering Purdue University West Lafayette Indiana USA

**Keywords:** additives, aqueous zinc‐metal batteries, charge‐transfer kinetics, hydrogen evolution reaction, multivalent batteries

## Abstract

Aqueous zinc metal batteries (AZMBs) are promising for next‐generation grid storage due to their low cost and intrinsic safety. However, their deployment is limited by poor zinc plating/stripping reversibility at the anode, driven by hydrogen evolution, corrosion, and dendrite formation. Solvation modulation of Zn^2+^ using high‐donor‐number additives is widely reported to enhance performance by imposing sluggish charge‐transfer and desolvation kinetics, often reflected in a reduced zinc electrodeposition exchange current (*i*_0,Dep_). Yet beyond a critical concentration, additive‐induced over‐suppression of deposition kinetics leads to pronounced anode instability, the mechanistic origin of which remains unresolved. Here, we establish a framework defining an optimal kinetic window for additive concentration. Using ZnSO_4_, ZnCl_2_, and Zn(OTf)_2_ electrolytes with systematically tuned solvation environments, we demonstrate that enhanced stability emerges from a synergistic interplay among electrodeposition, nucleation‐growth dynamics, and corrosion kinetics. Within this optimal regime, uniform restructured (002)‐oriented zinc deposition suppresses hydrogen evolution, improving reversibility and cyclability. Beyond this threshold, further reduction of *i*_0,Dep_ shifts the system toward mass‐transport induced corrosion‐dominated behavior, eliminating the restructuring advantage and accelerating hydrogen evolution. This framework is generalizable across additives that primarily alter Zn^2+^ solvation without significantly affecting mass transport. Collectively, these findings provide a rational basis for electrolyte design in AZMBs.

## Graphical abstract

Zinc anode performance is maximized within an optimal deposition‐kinetic window. Additives that tune the deposition exchange current density (*i*_0,Dep_) suppress HER at intermediate values, but overly fast kinetics promote dendrites and overly slow kinetics accelerate corrosion/HER. This mechanistic framework resolves the “optimal additive concentration” paradox in aqueous Zn batteries.

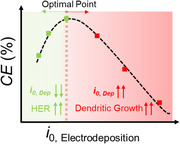

## 1 Introduction

The pursuit of reliable, sustainable, and efficient energy storage solutions has placed aqueous zinc‐metal batteries (AZMBs) at the forefront of modern electrochemical research in the battery community. With their abundant resources, inherent safety, and competitive energy densities (ranging from around 150 Wh/kg to 470 Wh/kg), AZMBs present a promising alternative to the state‐of‐the‐art lithium‐based chemistries [1, 2, 3, 4, 5, 6, 7, 8]. However, a critical challenge in the development of AZMBs lies in ensuring the stability of zinc metal anodes, which are prone to dendritic growth, and parasitic side reactions like hydrogen evolution reaction (HER) during charging (zinc plating on anode) of the AZMBs [9, 10, 11, 12, 13]. These issues not only degrade battery performance but also compromise long‐term reliability.

**FIGURE 1:**
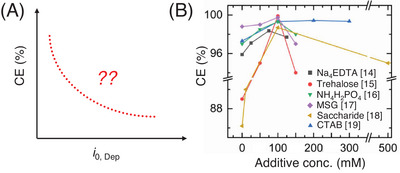
(A) Coulombic efficiency vs. charge transfer kinetics of zinc electrodeposition, showing theoretical expectation according to Sand's model and classical nucleation theory. (B) Coulombic efficiency vs. additive concentration plot for different additives, obtained from the literature, showing the optimal concentration of each additive.

In literature it has been shown that electrolyte composition is pivotal for optimizing zinc anode stability, and significant progress has been made through the addition of various electrolyte additives [14, 15, 16, 17, 18, 19]. The extensive literature on new electrolyte formulations/additive studies has provided a strong foundation for advancing electrolyte modification strategies and has significantly contributed to the development of high‐performing batteries [20, 21, 22, 23, 24]. These studies have shown that carefully crafted additive molecules can suppress side reactions, slow down the intrinsic charge transfer kinetics of zinc plating, decrease ionic conductivity, and enhance uniform zinc deposition morphology, offering substantial performance improvements. In the literature, these improvements in presence of an electrolyte additive are tied to one common experimental observable, sluggish charge transfer kinetics of zinc plating/lower exchange current for zinc plating/deposition kinetics/higher charge transfer resistance. Therefore, in principle, the schematic presented in Figure 1(A) should be the expected trend, where lowering exchange current of zinc deposition (*i*_0, Dep_) should always enhance the overall Coulombic efficiency (CE) or the stability of the anode. However, after a detailed analysis of literature pertinent to electrolyte additives in the field of AZMBs, it was realized that across the body of literature there is always an existence of an “optimal additive concentration,” beyond which the performance/anode stability diminishes. We note that the additive concentration in these works is inversely proportional to the charge transfer kinetics. As shown in Figure 1(B) and Figure S1, the CE values increase with increasing amount of additives, lowering the exchange current, but after adding a critical amount of additive/critical *i*_0,Dep_, the CE decreases. It was also realized that this observation was related primarily to additive chemistries that function by modulating the solvation matrix, along with a preferential adsorption of additives to the surface of the anode, which led to an overall sluggish charge trasnfer kinetics of zinc deposition and enhancement in the overall stability of AZMB. It must be noted that the majority of additive‐based studies in the literature focus on similar mechanistic pathways to enhance the performance of AZMBs. While these contributions have laid the groundwork for understanding additive behavior and maximizing the stability of AZMBs, the rationale behind the existence of the “optimal spot/critical *i*_0, Dep_ values” remains unexplored and unanswered.

A recurring finding in the field is that lowering the exchange current (*i*_0,Dep_) can simultaneously suppress HER, mitigate dendritic growth, and enhance battery longevity. Theoretically, according to Sand's model, reducing *i*_0,Dep_ should be beneficial as it decreases local current density, limiting mass transport‐induced dendritic instabilities [25]. Moreover, a higher *i*_0,Dep_ must promote rapid Zn^2^^+^ reduction, increasing the local current density and exacerbating dendrite growth due to mass transport limitations. These dendrites not only compromise battery longevity by increasing short‐circuit risks but also expose high‐surface‐area Zn deposits to parasitic side reactions [10, 26]. On the other hand, a low *i*_0,Dep_ suppresses side reactions by slowing Zn^2^^+^ reduction while also delaying dendrite formation by keeping the operational current density below the threshold (limiting current density) for dendritic instability. Moreover, classical nucleation and growth theory further suggests that a lower *i*_0,Dep_ promotes a higher nucleation density and smaller critical radius for deposited nuclei, thereby leading to more uniform zinc deposition [27, 28]. Under galvanostatic conditions, a reduced *i*_0,Dep_ leads to a higher nucleation overpotential, which promotes smaller critical nucleus radius and a high areal density of Zn nuclei. This enhances lateral growth, improving Zn morphology by minimizing localized protrusions that evolve into dendrites. Based on this overview, it is clear why slowing down deposition kinetics of zinc has emerged as the most promising solution in the additive literature to enhace anode stability. However, the critical limits of this approach has largely been overlooked in literature despite the observation of an “optimal additive concentration” for enhancing anode stability.

In this work, we report a mechanistic framework to understand the existence of an optimal additive concetration.  While reducing *i*_0,Dep_ does indeed suppress side reactions and delays dendritic growth, we show that excessively low *i*_0,Dep_ promotes side reactions and morphological instabilities, ultimately undermining long‐term performance. Through a systematic study involving temperature‐dependent experiments, model additive investigations, and a comparative analysis across common zinc salts (ZnCl_2_, ZnSO_4_, and Zn(OTf)_2_), we demonstrate that existing models such as Sand's model and classical nucleation theory fail to fully capture the observed trends. While these frameworks provide valuable insights into zinc deposition behavior, they overlook critical solvation kinetics, corrosion/side‐reaction kinetics, and interfacial effects that dictate anode stability in AZMBs. Our model accounts for these additional complexities and provides a comprehensive explanation for the seemingly unexplained decline in performance beyond the “optimal spot”. The findings presented in this work reveal the intricate interplay between zinc deposition kinetics, corrosion kinetics, mass transport, and morphological instabilities. To explain this observation, we combine temperature‐dependent kinetic analysis with ultramicroelelctrode (UME) electroanalysis, operando electrochemical mass spectrometry (ECMS), electrochemical quartz crystal microbalance (EQCM), and intermittent electrochemical impedance spectroscopy (EIS) during cycling. These complementary techniques enable direct quantification of parasitic hydrogen evolution, irreversible zinc accumulation, and interfacial charge‐transfer evolution, respectively. Together, they provide a unified, experimentally grounded picture linking additive‐induced kinetic modulation to zinc anode stability. Complementary Aurbach asymmetric cell testing and post‐cycling SEM imaging confirmed that the intermediate (“optimal”) additive concentration yielded the lowest capacity loss and minimal dead Zn formation, whereas both lower and higher concentrations produced increased residue, HER and surface passivation. Overall, this study provides a mechanistic understanding of the impact of electrolyte additives in AZMBs, emphasizing the limitations of conventional theoretical models and introducing a refined framework that better explains the interplay between charge transfer kinetics, mass‐transfer, corrosion kinetics, nucleation energetics, and dendrite suppression. Our findings offer crucial insights for designing next‐generation electrolyte formulations, bridging the gap between theoretical predictions and practical battery performance.

## 2 Results and Discussion

We begin with a theoretical construct to conceptualize how zinc electrodeposition kinetics influence the CE of AZMBs, considering the interplay between electrodeposition and side reactions, primarily HER. By establishing this foundational understanding, we aim to comprehensively evaluate the role of additives and their concentration in optimizing AZMB performance.

**FIGURE 2:**
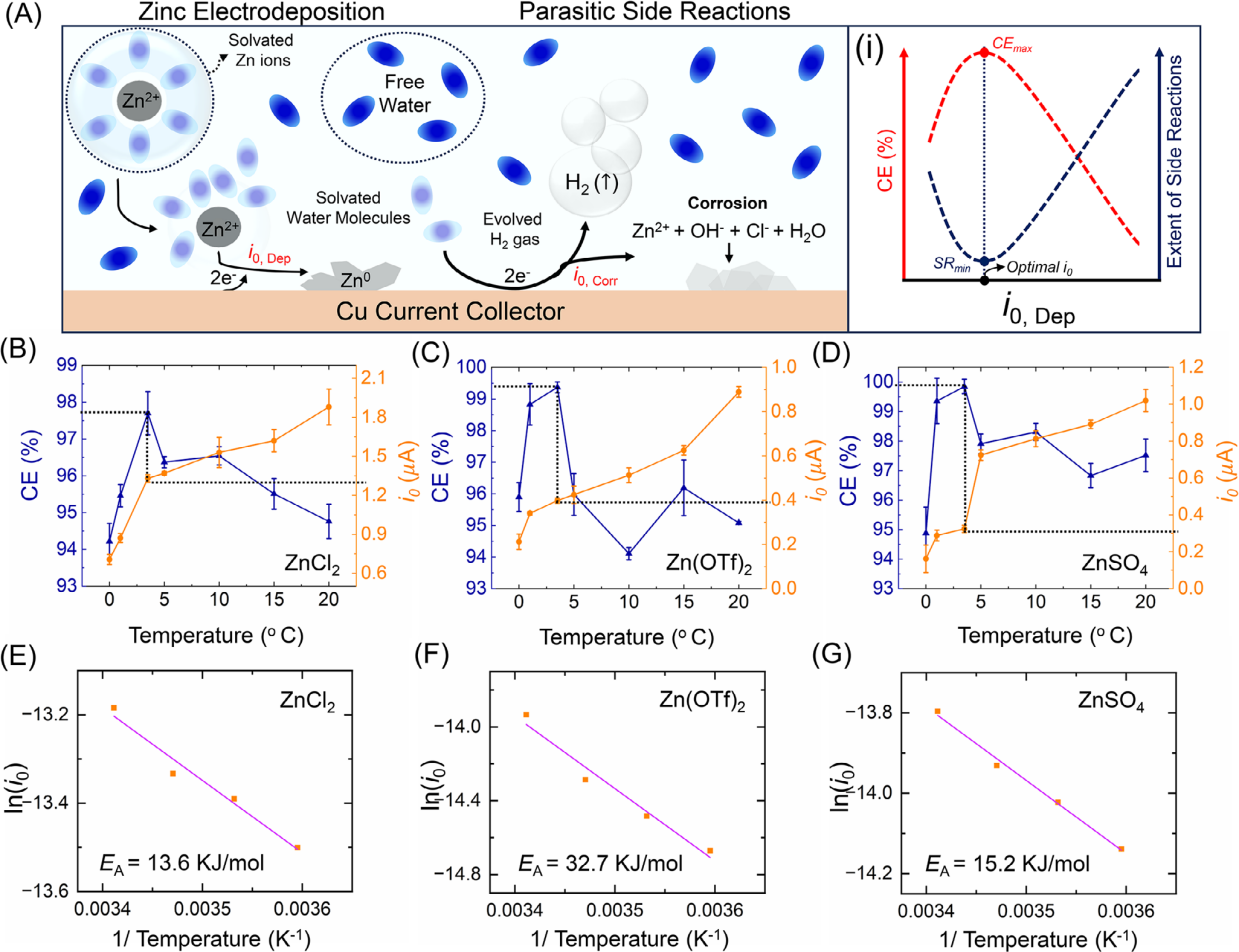
(A) Schematic representation of simultaneous zinc electrodeposition occurring along with hydrogen evolution and other side reactions. (i) Theoretical representation of the expected trend of coulombic efficiency and the extent of side reaction with the change in exchange current of zinc deposition (*i*_0, Dep_). (B) Effect of temperature on coulombic efficiency and exchange current in 1 M ZnCl_2_, (C) 1 M Zn(OTf)_2_ and (D) 1 M ZnSO_4_ electrolytes, representing the optimal exchange current values for the peak coulombic efficiency (results represent data from three independent experiments for each electrolytes (Figure S27)). (E) Arrhenius style plot for ZnCl_2_, (F) Zn(OTf)_2_, and (G) ZnSO_4_ electrolytes.

Building on existing literature in the AZMB field, it is well reported that slowing down charge transfer kinetics can enhance the cyclability of AZMBs [14, 15, 16, 17, 18, 19, 20, 21, 22, 23, 24]. This raises a fundamental question: Is there a limit to how much the charge transfer kinetics can be slowed down, or does CE continue to improve indefinitely as charge transfer becomes progressively slower? To begin, let's consider the processes occurring within AZMBs during zinc electroplating (see Figure 2(A)). As zinc ions diffuse toward the current collector under an applied bias, they are solvated by water molecules, which comprises the solvation matrix. Prior to electrodeposition, the water molecules in the solvation matrix undergo reorganization and adsorption, allowing the zinc ions to accept electrons from the current collector. This rearrangement is essential because the system needs to reach a new equilibrium configuration for charge transfer to occur efficiently. The reorganization energetics are associated with the desolvation kinetics, which have been shown to be intimately tied to the long‐term stability of the anode. The overall process of ion desolvation followed by electron transfer is collectively reported as charge transfer of zinc ions. A simplistic interpretation of this idea is presented in the schematic presented in Figure 2(A). The following discussion holds for galvanostatic plating of zinc ions which is representative of AZMB operating conditions. Under an applied reduction current, the solvated zinc ion diffuses toward the electrode surface. The water molecules involved in the solvation matrix are referred to as solvated water molecules, whereas free water molecules comprise the bulk water network. As the solvated zinc ion approaches the electrode surface, the solvated water structure reorganizes to facilitate charge transfer, enabling the electrodeposition of zinc onto the current collector. As demonstrated by previous work from our group and other studies in the literature, during this process, solvated water molecules are prone to undergoing HER alongside electroplating of zinc [12, 13, 29, 30, 31, 32, 33, 34]. Additionally, the counter anions of the electrolyte and water molecules may participate in parasitic side reactions/HER induced precipitation reactions, leading to the formation of zinc hydroxide‐containing corrosion products or other components of the solid electrolyte interphase (SEI) [3, 35, 36, 37]. These corrosion products may be insulating in nature and pose a significant challenge to the long‐term stability of anodes in AZMBs.

The kinetics of these reactions are represented by exchange currents, specifically *i*_0,Dep_ and *i*_0,Corr_, as shown in Figure 2(A). Here, *i*_0,Corr_ is used as an effective kinetic descriptor representing the combined rate of parasitic zinc‐consuming processes occurring during deposition. This includes both electrochemical HER involving interfacial electron transfer and chemically driven zinc corrosion reactions that proceed without direct charge transfer. While the latter does not strictly obey Butler‐Volmer kinetics, its rate remains coupled to interfacial transport and surface kinetics; thus, *i*_0,Corr_ provides a useful phenomenological parameter for quantifying total anode loss and hydrogen evolution under different kinetic regimes. It is widely accepted in the literature that slowing down the charge transfer kinetics (*i*_0,Dep_) improves overall anode stability and suppresses side reactions, leading to higher CE [14, 15, 16, 19, 23, 38, 39, 40]. Thus, to enhance the cyclability and CE of AZMBs, it is crucial to control the kinetics of both the charge transfer and side reactions (*i*_0,Dep_ and *i*_0,Corr_, respectively). We hypothesize that balancing these processes is essential for optimizing anode stability. Specifically, the widely accepted idea of slowing down charge transfer to maximize stability is incomplete without considering side reaction kinetics. An excessive reduction in *i*_0,Dep_ can increase the propensity of side reactions, leading to poor cyclability/anode stability. This is especially critical under galvanostatic conditions, where the charge transfer must occur at a fixed rate depending on the value of applied current/specified rate. This hypothesis is illustrated in Figure 2(A)(i), where CE improves as *i*_0,Dep_ is slowed down, up to a certain threshold. Beyond this point, the increased propensity for side reactions leads to a decline in CE. This framework effectively explains the existence of an optimal additive concentration, which maximizes the stability and efficiency of AZMBs. At the same time, if sluggish kinetics always increase side reactions, then lowering *i*_0__,Dep_ would never improve anode stability, which contradicts experimental observations. In reality, slowing zinc‐deposition kinetics can also promote morphological restructuring of deposited Zn, which further suppress corrosion rates. Therefore, a holistic picture that integrates the kinetics of deposition and corrosion with kinetics‐coupled morphological restructuring is essential, as discussed in the following sections.

This perspective is directly relevant in the context of additives reported to exhibit a “sweet spot” or “optimal concentration” (see Figure 1), where cycling performance is maximized by suppressing charge transfer kinetics. Beyond this optimal concentration, the behavior degrades as side reactions begin to dominate, overshadowing the beneficial effects of slowed charge transfer kinetics. Based on this conceptual understanding, if the core principle for better AZMB anode stability is achieving slower (lower *i*_0,Dep_) kinetics to a certain extent, this should be achievable through methods beyond additive concentration optimization. For example, we hypothesize that modifying external factors, such as temperature, could yield a similar trend in CE values. Temperature is a crucial parameter that can readily modify zinc and its byproducts’ morphology as well as control their formation kinetics [41, 42, 43, 44, 45]. Therefore, in the following section, we first explore the utilization of temperature as a parameter to tune charge transfer energetics, providing an illustration of the hypothesis presented earlier. According to the Arrhenius equation [46, 47], the rate of the reaction is given by Equation (1):

(1) $$k=Ae^{-\frac{E_{a}}{RT}}$$

(2) $$k=\frac{i_{0}}{FAC}$$

(3) $$\ln\;i_{0}=\ln\left[A^{\prime }\right]-\left[\frac{E_{a}}{R}\right]\frac{1}{T}\;$$

where *k* is the rate constant, *A* is the pre‐exponential factor, *E_a_* is the activation energy, *R* is the gas constant, and *T* is the temperature. Based on Equation (1), the reaction rate constant, which dictates the charge transfer kinetics, can be tuned by modulating the temperature. Charge transfer kinetics is related to the rate constant according to Equation (2), where *F* is the Faraday's constant, *A* is the surface area of the electrode and *C* is concentration of the species. By combining Equations (1) and (2), we get Equation (3) which shows the modified version of the Arrhenius equation that relates charge transfer kinetics with the activation energy, where A’ is a constant of the multiplication product of Faraday's constant, pre‐exponential factor, surface area and concentration. This prompted us to conduct low‐temperature experiments to promote slower charge transfer kinetics. Figures 2(B)‐(D) shows the temperature effect on CE and *i*_0,Dep_ values determined using UMEs for commonly utilized 1 M ZnCl_2_, Zn(OTf)_2_, and ZnSO_4_ electrolytes for AZMBs. The Arrhenius style plots shown in Figures 2(E)‐(G) represents the linear fit which further suggested the lowering of *i*_0,Dep_ with lower temperature. It also allowed us to calculate the activation energies of the three different zinc electrolytes, which shows that Zn(OTf)_2_ has the highest activation energy followed by ZnSO_4_ and finally ZnCl_2_. Temperature‐dependent electrochemical impedance spectroscopy (EIS) measurements further corroborate the kinetic trends extracted from Tafel analysis (*i*_0_ values at all temperatures). As shown in Figures S2, S3, the charge transfer resistance (*R*_ct_) decreases systematically with increasing temperature for all electrolytes, consistent with the expected Arrhenius‐type activation of zinc deposition kinetics.

**FIGURE 3:**
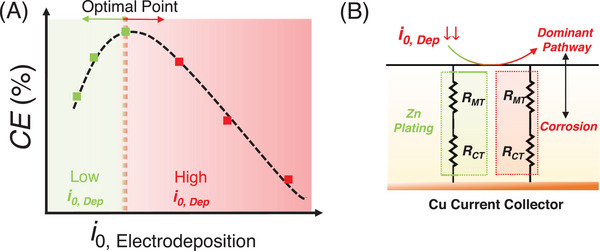
(A) Schematic showing how coulombic efficiency peaks at the optimal zinc electrodeposition kinetics (*i*_0,Dep_) and how performance degrades before or after this point. (B) Schematic representation of possible pathways of the electrons to flow through either Zn plating or corrosion resistance, depending on the zinc deposition kinetics (*i*_0,Dep_). Here, *R*_MT_ denotes the mass‐transport resistance associated with interfacial ion transport. On the plating pathway, *R*_MT_ reflects limitations in Zn^2^^+^ replenishment when *i*_0,Dep_ becomes too low, while on the corrosion pathway, *R*_MT_ describes restricted transport of proton‐donating species (H_2_O/H_3_O^+^) or soluble corrosion intermediates.

The CE and *i*_0,Dep_ values were calculated using fast‐scan cyclic voltammetry (FSCV) with UMEs. This approach was adopted because FSCV, as reported previously by our group and others, enables accurate determination of charge transfer kinetics [48, 49, 50]. By minimizing mass transfer effects, FSCV ensures that the calculated *i*_0,Dep_ values truly reflect the intrinsic kinetics of the electrodeposition process, making it an indispensable tool for our analysis. Interestingly, a clear trend is observed across all three electrolytes: lowering *i*_0,Dep_ by reducing the temperature initially leads to an increase in CE, which ultimately peaks and then deteriorates if the temperature or *i*_0,Dep_ is further reduced. This certain temperature and the certain *i*_0,Dep_ suggests an “optimal point” for the different counter anions where the CE peaks. For all of the counter anions, the optimal points were observed at similar temperature with different values of *i*_0,Dep_. We hypothesize that this occurs solely due to increased propensity for side reactions at lower temperatures being the critical threshold. According to Sand's model, lowering *i*_0,Dep_ is beneficial for delaying the onset of dendrite formations while higher value of *i*_0,Dep_ promotes dendrite formation. So, the observed optimal point for the highest CE is also true from the dendrite formation argument. However, we hypothesize that the corrosion rates decrease upto the optimal point due to favorable morpholgical retexturing as a result of sluggish charge trasnfer kinetics. This aspect is discussed in later sections. Overall, this trend provides compelling evidence for the hypothesis proposed earlier, affirming the existence of a “sweet spot” in charge transfer energetics where optimal cycling performance is achieved. This trend is further simplified to show the relationship between CE and *i*_0,Dep_ in Figure 3(A), where it is shown that decreasing *i*_0,Dep_ beyond a critical value will result in lower CE.

This may cause *i*_0,Corr_ to dominate rather than *i*_0,Dep,_ since the side reactions would be the preferred pathway for the electron provided by the electrode. Another intuitive way to understand this can be explained as if a cathodic current is incident on a metal current collector, the total current, *i*_c,tot_, would be equal to the sum of all cathodic currents that are possible (i.e., all molecules that are willing to accept electrons). At low overpotential approximation leading to no mass transfer effects and assuming there are two pathways for the electrons to go (Figure 3(B)), a mathematical expression can be described as Equation (4),

(4) $$i_{c,tot}=i_{0,Dep}\;e^{-\alpha f\eta_{Dep}}+i_{0,Corr}e^{-\alpha f\eta_{Corr}}$$

where, α is the transfer coefficient (assumed to be 0.5), *f* is the inverted thermal voltage and *η* is the overpotential [13, 51]. Also, the *i*_0,Corr_ corresponds to the summation of all the side reactions that may occur during the electrodeposition process. Now from this equation, if we achieve a condition where we make the *i*_0,Dep_ too low, then the total cathodic current would be dominated by *i*_0,Corr_. This would make the charge transfer resistance (*R*_ct_) for zinc electrodeposition so high that the only possible pathway for the electron would be the parasitic side reactions. This analysis is true for the extreme cases when deposition kinetics are really sluggish (i.e., beyond the optimal thresold). This can be visualized from the equation (5), which is obtained under low overpotential approximation of the Butler‐Volmer equation [51],

(5) $$R_{ct}=\frac{RT}{Fi_{0}}\;$$

where, *R* is the gas constant, *T* is the temperature, *F* is the Faraday constant and *i*_0_ is the exchange current. The inverse proportionality of *R*_ct_ and *i*_0_, explains the Figure 3(B) and the dominant behavior of side reactions when *i*_0,Dep_ becomes too low. Also, note that our previous studies and literature have shown that the HER occurs dominantly from the solvated water molecules [12, 13, 29, 30, 31, 32, 33, 34]. Based on these findings, we can hypothesize that leading up to the optimal point, relatively sluggish charge transfer kinetics promote more homogeneous and high‐coverage zinc deposition with a “suitable” morphological orientation (discussed later), which in turn suppresses side reaction/HER kinetics. This results in a synergistic effect: suppressed hydrogen evolution and more uniform plating. However, beyond a critical threshold, if the deposition kinetics become excessively slow and mass transport limitations exist, parasitic side reactions begin to dominate, offsetting the morphological advantages. Using this framework, we next explore how the hypothesis unfolds in the context of electrolyte additives that slow down charge transfer kinetics.

**FIGURE 4:**
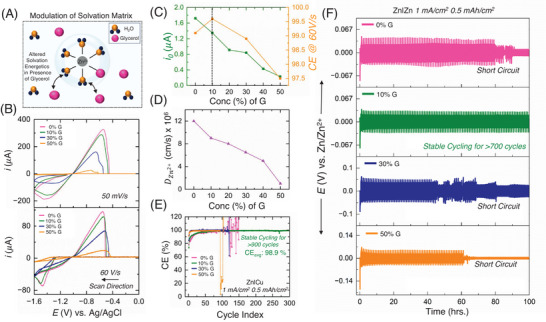
(A) Schematic showing the solvation matrix of zinc ion being affected by glycerol molecule. (B) Overlay of cyclic voltammograms of 1 M ZnCl_2_ with different concentrations of glycerol on tungsten ultramicroelectrode at the scan rate of 0.05 V/s and 60 V/s. (C) Double y‐axis plot showing the trend of *i*_0_ and coulombic efficiency (obtained from the fast scan voltammogram) with different concentrations of glycerol. (D) Diffusion coefficient ($D_{Zn^{2+}}$) of Zn^2+^ in presence and absence of different concentrations of glycerol in 1 M ZnCl_2_. (E) Comparison of Zn|Cu asymmetric cell data for 1 M ZnCl_2_ electrolyte with different concentrations of glycerol. (F) Zn|Zn symmetric cell cycling at a current density of 1 mA/cm^2^ and a capacity of 0.5 mAh/cm^2^ in 1 M ZnCl_2_, comparing results with different concentrations of glycerol.

We selected glycerol (G) as the additive to tune the solvation structure of the bare electrolyte (1 M ZnCl_2_) due to its simplicity, low cost, and unique molecular structure featuring three hydroxyl groups. Glycerol is a naturally viscous liquid that is highly miscible with water and already reported as an electrolyte additive for improving AZMB anode stability and performance [52, 53, 54, 55, 56, 57, 58, 59, 60, 61], making it an ideal choice for our study. We prepared three concentrations of glycerol in a 1 M ZnCl_2_ aqueous solution: 10% (w/w), 30% (w/w), and 50% (w/w). Using these electrolytes, we tested the performance of symmetric and asymmetric coin cells to evaluate the effect of additive concentration. Figure 4(A) shows how glycerol partially replaces the water molecules in the Zn^2+^ solvation matrix and modulates desolvation kinetics to slow down the charge transfer kinetics. ^67^Zn NMR results shown in Figure S4, suggest a downfield shift when glycerol was introduced in the electrolyte. Therefore, the glycerol molecule alters the solvation shell of Zn^2+^ ion, which ultimately affects the charge transfer kinetics. To understand the existence of an optimal concentration of the glycerol additive, it is essential to examine the charge transfer kinetics of zinc metal electrodeposition as a function of glycerol concentration in the electrolyte. Additionally, the mass transfer of Zn^2^^+^ ions toward the electrode surface must be considered, as this process is significantly influenced by changes in electrolyte viscosity (increases with glycerol concentration). In our previous study, we demonstrated how fast scan kinetic analyses using UMEs can be employed to measure key kinetic parameters, such as the diffusion coefficient (*D*_Zn_^2^^+^) of Zn^2+^ ions and exchange current (*i*_0_) [49, 50].

The cyclic voltammetry (CV) of zinc electrodeposition on a tungsten UME Figure 4(B) was studied using a two‐electrode setup with an Ag/AgCl reference/counter electrode. At a slow scan rate (0.05 V/s), zinc deposition began around ‐1.1 V, reaching a diffusion‐limited peak near ‐1.4 V, followed by a characteristic nucleation loop and a stripping peak during the reverse scan. Faster scan rate voltammetry (60 V/s) showed charge‐transfer‐controlled deposition, as mass transfer effects were negligible (Figure S5). Details of scan rate dependent study of zinc electrodeposition have been provided in the SI (Figures S5, S6, S25, and S26). The impact of glycerol as an additive was examined, revealing that increasing concentrations reduced the stripping peak current due to decreased deposition charge (*Q*_Dep_), as shown in Table S1. This indicates sluggish charge transfer kinetics at higher additive concentrations. Diffusion coefficients calculated from forward‐sweep CVs Figure 4(D), (Figure S7) confirmed that increasing glycerol concentration significantly reduced Zn^2^^+^ diffusion coefficient values. Exchange currents (*i*_0_) were determined via Tafel analysis (Figure S8), and CE values were extracted from FSCVs Figure 4(C) to minimize mass transfer effects. Furthermore, EIS measurements reveal a systematic increase in *R*_ct_ with glycerol concentration (Figure S9), rising from 10.1 to 17.1 Ω·cm^2^ between 0% and 50% G. The monotonic increase in *R*_ct_ mirrors the decrease in *i*_0,Dep_ extracted from Tafel analysis on UMEs, reinforcing that glycerol slows zinc deposition by increasing interfacial kinetic barriers associated with solvent reorganization and ion transport. These results demonstrate how glycerol concentration influences zinc deposition kinetics and CE. Similar to the cases outlined in Fig. 1, an “optimal spot” was realized at 10% G, where CE was maximum. Further increasing the glycerol concentration led to a decrease in both CE and exchange current values.

From Figure 4(C), we can see the same trend visualized at Figure 3(A), that the CE is maximum at a certain concentration (10% G) and afterwards degrading with increasing additive concentration. Figure 4(E) illustrates the asymmetric Cu|Zn cell performance for all the electrolytes, highlighting the CE, a key indicator of practical battery performance, across varying glycerol additive concentrations. As expected from the FSCV CE results, the 10% G cell demonstrated the best performance, achieving approximately 900 cycles (Figure S10) with a stable average coulombic efficiency (ACE) of 98.9% and with a stable charging/discharging profile (Figure S11). With increasing glycerol content, the cells initially exhibited high coulombic efficiencies, but their performance rapidly declined, leading to short circuits around the 100^th^ cycle. Similarly, at 0% G, the absence of additive and unsuitable reorganization kinetics failed to sustain long‐term stability, with asymmetric cells also short‐circuiting after ∼100 cycles. Figure 4(F) illustrates the symmetric Zn|Zn coin cell performance across varying glycerol concentrations. As expected, 10% G cell exhibits smooth and symmetric plating/stripping profile, which is absent in the 0%, 5%, 30% and 50% G cells (Figure S12). While the nucleation overpotential (*η*_nucleation_) and polarization voltage (α) are nearly identical for both 0% and 10% G cells, the polarization voltage of the 0% G cell continuously fluctuates (Figure S13), ultimately leading to a short circuit at around 80^th^ cycle. In contrast, the 10% G cell maintained stable plating‐stripping behavior for approximately 700 cycles. Beyond 10% G, cell performance deteriorated sharply with increasing G contents in the electrolyte. As shown in Figure 4(F), both the 30% and 50% glycerol cells experienced short circuits by the ∼60th cycle. The 30% G cell displayed unstable plating and stripping behavior, while the 50% G cell, despite initially exhibiting promising stripping and plating characteristics and the highest *η*_nucleation_ (attributable to its high viscosity and lowest *i*_0_ value), failed prematurely. Importantly, the same “optimal concentration” behavior persists under high current density (10 mA cm^−^^2^) and high areal capacity (10 mAh cm^−^^2^), indicating that the proposed mechanistic framework remains valid under aggressive cycling conditions (Figure S14).

Despite reduced *i*_0,Dep_ in higher content of G cells, the performance degraded significantly, a phenomenon not fully captured by Sand's model and classical theories. Only by accounting for solvent reorganization kinetics, morpholocial texturing, and side reaction suppression leading up to the optimal condition arguments from our proposed model, we can explain this paradox. These results clearly identify the 10% G cell as the best‐performing configuration, delivering stable charging and discharging behavior alongside the longest lifespan according to symmetric‐cell data. Although Zn NMR indicates that glycerol perturbs the Zn^2^^+^ solvation shell, the maximum CE occurs at an intermediate diffusion coefficient rather than at the lowest diffusivity. At this stage, this suggests that the optimal glycerol concentration reflects a balance between solvation‐modified charge‐transfer kinetics and mass‐transport limitations, rather than dominance by either effect alone. To further clarify this point, let's take a step back and try to understand how glycerol or any other additive is working on the electrolyte environment to produce the optimal concentration effect on CE values. Glycerol alters the solvation matrix of zinc ion and also decreases the diffusion of zinc ions by introducing viscosity. Thus, a key question is whether glycerol (or any additive) enhances anode stability primarily by affecting desolvation kinetics or by imposing viscosity driven mass transport limitation, or by both mechanisms simultaneously. This can only be answered when we can exclude the alteration of solvation matrix by the additive but only have the mass transfer limiting effect. Moreover, understanding this is really important to ensure there are no mass trasnfer effects leading to the enhanced anode stability at the optimal concentration.

To address this, we employ a model additive to disentangle the effects, allowing us to isolate and examine glycerol's role in altering mass transfer and charge transfer processes, which, in turn, will help us understand the observed trends in CE. To demonstrate this, we chose a different molecule as a mass transfer limiting agent, polyvinylpyrrolidone (PVP), also known as a “crowding agent” due to its huge molecular structure. This molecule has been shown to only affect the mass transfer of zinc ions but doesn't interfere with the solvation matrix of zinc ions. Mullins et al. have demonstrated the use of this molecule as an electrolyte additive in order to reorient the orientations of zinc for smoother deposition, leading to a better stability of the zinc anode [62]. It is important to recognize that the introduction of a new additive, PVP, which primarily affects mass transfer, is intended to isolate and understand the influence of viscosity on the mass transfer profile. This insight from PVP will provide a clearer understanding of glycerol's dual role: altering solvation dynamics and inducing sluggish mass transfer with increasing glycerol concentration.

**FIGURE 5:**
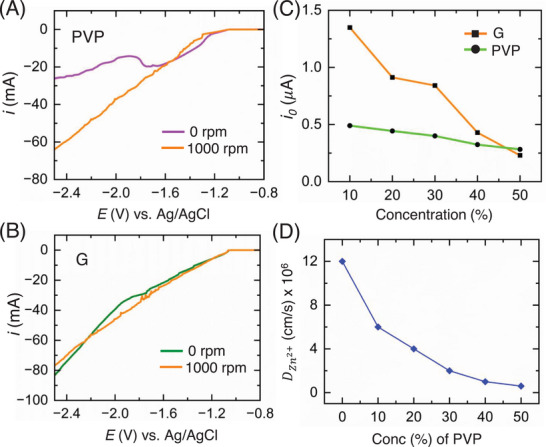
(A) Linear sweep voltammograms of 1 M ZnCl_2_ solution with 10% polyvinylpyrrolidone (PVP) and (B) 10% G using the Rotating disk electrode setup. (C) Exchange current (*i*_0_) vs. concentration plot for PVP and glycerol, in 1 M ZnCl_2_. (D) Diffusion coefficient ($D_{Zn^{2+}}$) of Zn^2+^ in presence and absence of different concentrations of PVP in 1 M ZnCl_2_.

To characterize the mass transfer limiting effect of PVP, we did a separate rotating disk electrode (RDE) experiment, where a glassy carbon macroelectrode (3 mm dia.) was used as the rotating working electrode, Zn foil as a counter electrode and an Ag/AgCl in 1 M KCl as the reference electrode. To determine whether the additives induce mass transfer limitations, we compared the linear sweep voltammetry (LSV) curves with and without electrode rotation. Ideally, if the electrolyte primarily affects solvation energetics, we expect no significant effect from rotating the electrodes. In contrast, if the electrolyte impacts mass transfer, we anticipate notable differences in the polarization curves due to the electrode rotation [51, 62]. As shown in Figure 5(A), we can observe the significant difference in the polarization curves with and without rotation for 10% (w/w) PVP electrolyte. The sudden dip in the LSV curve at 0 rpm indicates the mass transfer limitation induced by PVP, since this dip is lost in the 1000 rpm curve as the rotation can eliminate the mass transfer limitation. Whereas, Figure 5(B) shows the LSV curves with and without rotation for 10% G electrolyte, which is also very similar to the LSV curves with and without rotation found for 0% G electrolyte (Figure S15). These results suggest that glycerol indeed alters the solvation shell along with increasing viscosity while PVP only introduced the crowding effect or viscosity to the electrolyte without altering the charge transfer profiles. The key takeaway here is that, despite changing viscocity of the electrolyte due to addition of glycerol, the electrochemistry in presence of glycerol is primarily dictated by the solvation enegetics of zinc up to the optimal point, unlike the PVP‐containing electrolytes, which are mass transport dominated. Herein, we speculate that up to a threshold concentration of glycerol, solvation energetics governs the electrochemical performance, wheares beyond that threshold, mass transport limitations become signficant (compare diffusion coefficient values for zinc ions for glycerol and PVP shown in Figure 4(D) and 5(D)).

Figure 5(D) shows the diffusion coefficient values decreasing with the increasing concentration of PVP in the electrolyte. Compared to glycerol, PVP increases the viscosity in a much faster rate as the *D*_10%_ for PVP is almost similar to *D*_30%_ for glycerol (refer back to Figure 4(D)). This is indicative of the crowding effect of the PVP molecule while glycerol affects the solvation shell along with increasing the viscosity of the electrolyte (sluggish mass transfer). Based on this, we expect that higher concentrations of glycerol can have dual role i.e., modifying solvation and inducing mass transport limitations like PVP. Additionally, we observed that the exchange current values of kinetically limited zinc electrodeposition determined using FSCV for PVP with different concentrations were almost similar while the exchange current values changed significantly with different concentrations of glycerol (Figure 4(C)). Note that, this shows the accuracy of the FSCV technique, whereas the traditional methods would not be able to accurately capture this since they would be convoluted by the kinetics involved with SEI formation. This clearly shows that the PVP molecule did not affect the solvation shell of Zn^2+^ ion, hence had no significant effect on changing the charge transfer kinetics/exchange current of the reaction. On the contrary, in the presence of glycerol the exchange current values decreased as a function of concentration which shows the ability of glycerol to primarily modulate the solvation energetics and dominate the interfacial kinetics at moderate concentrations. This comparison between glycerol and PVP as a function of concentration highlights that, changes in electrolyte viscosity cannot be simplistically attributed solely to mass transfer effects. In essence, it demonstrates that glycerol not only alters the mass transfer profile but also significantly impacts the solvation matrix of Zn^2^^+^ ions. Mullins et al. found that 40% (w/w) PVP performed the best in terms of cyclability of coin cells [62] which aligns with our results with PVP where 50% (w/w) PVP performed the best as an electrolyte additive in 1 M ZnCl_2_ electrolyte. The coin cell cycling data for symmetric configurations can be found in Figure S16. It is important to note that with PVP, performance improved consistently with increasing viscosity or PVP concentration in the electrolyte. That being said, it should be noted that if the concentration is too high (60% PVP (w/w)) to essentially reach the point of no ionic mobility due to extreme viscosity, the overall performance would decline. Nevertheless, this trend contrasts with the results observed for glycerol, where an optimal concentration was identified. This difference is attributed to glycerol's dual effect: primarily altering the solvation matrix alongside its impact on mass transfer at higher concentrations. Furthermore, we may infer that the optimal additive concentration trend would only be valid when the additive only slows down the *i*_0,Dep_ but not affect the mass transfer phenomenon, which is the most common scenario in literature. It should be mentioned that this does not mean that only the charge transfer kinetics is important. We must also consider mass transfer kinetics and find that critical balance between these two to optimize the electrolyte/additive design [49]. In most cases, the charge transfer kinetics is only being considered, however, the balance between charge transfer and mass transfer dictates the overall performance of the electrolyte/additive in zinc electrodeposition. It is important to note that the existence of an optimal point, as reported in this study, is primarily applicable to additive chemistries that modulate solvation energetics without significantly affecting mass transport characteristics like glycerol. Therefore, careful consideration of mass transport effects is essential using tools like RDE when evaluating the true role of additive chemistries in optimizing zinc anode performance.

**FIGURE 6:**
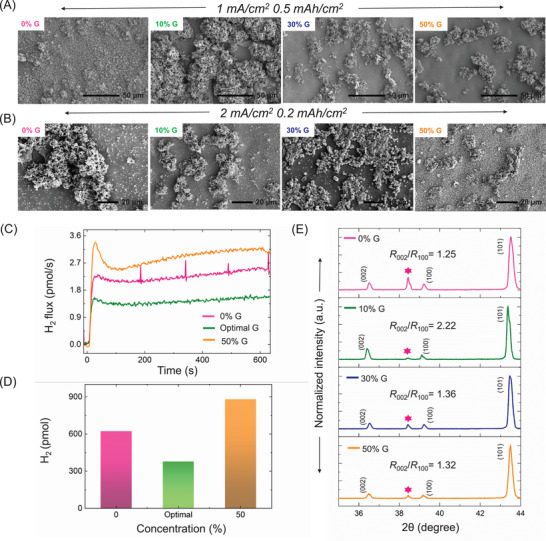
(A) Scanning electron microscopy (SEM) images of zinc electrodeposition on Cu foil in different glycerol concentration at the same current density and capacity as the coin cells. (B) SEM images of zinc electrodeposition on Cu foil in different glycerol concentration at higher current density (2 mA/cm^2^) and lower capacity (0.2 mAh/cm^2^). (C) Electrochemical mass spectrometry data corresponding for hydrogen flux in 0%, optimal, and 50% G. (D) Bar plot displaying the amount of evolved hydrogen gas in different concentration of glycerol. (E) XRD patterns of electrodeposited Zn on Cu foil for different glycerol concentrations. For the XRD measurements, deposition of Zn on Cu foil was done in the same current density and capacity of the coin cells. The pink starred reflection is caused by the exposed Cu surface since our substrate is Cu foil. An XRD pattern of a bare Cu foil provides the same reflection (Figure S21). This particular reflection arises from thin layer of copper oxides (JCPDS/PDF No. 05–0667 and JCPDS/PDF No. 45–0937).

In this section, we demonstrate that, up to the optimal additive concentration, a synergistic interplay between nucleation‐growth kinetics and solvation dynamics drives uniform zinc deposition with preferential crystallographic orientations that are intrinsically less prone to corrosion. Beyond this optimum, mass transport induced corrosion becomes dominant and negates the restructuring advantage. Here we show how the *i*_0,Dep_ vs. CE trend correlates with the observed morphology of electrodeposited zinc and extent of side reactions which are crucial for achieving long‐term stability. Figure 6(A) represents the SEM images of electrodeposited zinc on Cu current collector in different glycerol electrolytes. 0% G gave flake‐like morphology with almost no cluster‐like zinc deposition, whereas 10% G yielded a cluster‐like morphology with the highest surface coverage, indicating a smoother deposition. Importantly, visually uniform morphologies in additive‐free electrolytes may arise from homogeneous coverage by corrosion products (e.g., Zn(OH)_2_) formed via accelerated HER and chemical corrosion, rather than from uniform Zn^0^ deposition. Suppressing these side reactions with additives promotes more selective metal growth, even if the initial morphology appears less uniform. On the contrary, 30% G and 50% G gave uneven deposition with distributed small clusters. Since these small clusters are very distributed, formation of dendrites and dead zinc during prolonged cycling may increase. To further probe interfacial stability during operation, EIS was performed intermittently during cycling for different glycerol concentrations (Figure S17). In the glycerol‐free electrolyte (0% G), the Nyquist response evolves significantly with cycle number, accompanied by a progressive increase in *R*_ct_, indicative of continuous surface roughening, dead‐Zn accumulation, and interfacial degradation. At high glycerol contents (30% and 50% G), *R*_ct_ also increases markedly during cycling, reflecting excessive transport resistance and unstable interfacial evolution under over‐suppressed deposition kinetics. In contrast, the optimal glycerol concentration (10% G) exhibits the smallest change in *R*_ct_ and minimal distortion of the impedance profile across cycles, demonstrating a stable zinc‐electrolyte interface. This result provides direct operando evidence that the optimal kinetic window maximizes CE via minimizing surface evolution during repeated zinc plating/stripping. Figure 6(B) illustrates the evolution of zinc deposition morphology under high current density and low‐capacity conditions. As the glycerol content increases, the nucleation overpotential of zinc also rises, leading to a decrease in cluster diameter (see Figures S18, S19). This trend suggests that higher glycerol concentrations facilitate the formation of smaller critical nuclei, which—according to classical nucleation and growth theory—should promote smoother deposition and improved battery performance. However, kinetic analysis and coin cell performance data reveal a contrasting outcome: there exists an optimal level of additive concentration and corresponding charge transfer suppression that yields the best performance. This deviation arises because conventional models do not consider the dynamics of side reactions, which are critically addressed in this work. We speculate that while higher glycerol concentrations may promote smaller critical nuclei, the resulting high surface area and increased catalytic activity could enhance parasitic side reactions. The extent and implications of these side reactions are discussed in the following section. Most importantly, Figure 6(C) presents the electrochemical mass spectrometry (ECMS) data, which strongly supports our hypothesis regarding the optimal glycerol concentration. The hydrogen flux was at its lowest at this optimal concentration, reinforcing the correlation between *i*_0,Dep_ and suppressed side reactions. As expected, deviations from this optimal point ‐ both lower and higher glycerol contents ‐ led to significantly increased hydrogen evolution. The transient spikes in the H_2_ flux (notably in the 0% G electrolyte) are attributed to microbubble nucleation and detachment/rupture events during vigorous HER, which intermittently release trapped gas into the ECMS flow stream. The reduced frequency of such spikes at the optimal glycerol concentration is consistent with suppressed HER and more stable interfacial gas evolution. Notably, the 50% G electrolyte exhibited even higher hydrogen flux than 0% G, indicating that excessive glycerol content accelerates side reactions rather than mitigating them. This is further corroborated by Figure 6(D), where the amount of HER follows the same trend, with 50% G displaying the highest HER, suggesting that at this concentration, side reactions dominate due to the severely reduced *i*_0,Dep_ alongside mass transport limitations. Additionally, Figure S20 represents XRD patterns at low angles where zinc reflections are not expected. Supporting the electrochemical findings, distinct patterns were observed for both 0% and 50% G, suggesting the existence of side products such as HClO_4_.xH_2_O and Zn_5_(OH)_5_Cl_2_.H_2_O [63]. Whereas negligible reflections appeared for 10% G, further validating our conclusion. These findings highlight the critical role of optimizing *i*_0,Dep_ to minimize side reactions, as reflected in the ECMS results.

Figure 6(E) and Figure S22 show the XRD patterns of deposited zinc in different glycerol and PVP electrolytes, respectively. In the literature, the intensity ratio of the (002) to (100) orientation has been established as a reliable metric for evaluating the nature of electrodeposition [19, 20, 23, 62, 64, 65, 66]. A higher ratio is indicative of more homogeneous deposition and suppressed HER kinetics which favors the long‐term cyclability of coin cells. Figure S23 shows the evolution of *R*_002_/*R*_100_ as a function of glycerol concentration, where the ratio peaked at the optimal point and declined later on. Let's first focus on Figure S22, where the relative intensity ratio between (002) and (100) planes decrease with increasing PVP concentration. The mass transfer limitation effect of PVP inhibits the (002) orientations allowing the crystallographic reorientation of Zn deposition to expose the (100) and (101) textures, which undergo a “nucleation‐merge‐growth” process to form a uniform and compact Zn deposition [62]. Note that PVP did not affect the solvation matrix of zinc ions and hence did not affect the charge transfer kinetics (refer to Figures 5(A),(C) while glycerol does. As demonstrated by Mullins et al., these results, while contradicting glycerol's trend, that a higher (002) to (100) intensity ratio indicates superior performance, reveal an opposite trend in the case of PVP. Here, the enhanced performance arises from the controlled growth of the high‐energy (101) and (100) orientations. Notably, different additives investigated in this work (e.g., glycerol versus PVP) promote distinct and even opposite orientation trends while still improving zinc anode performance, highlighting that crystallographic texture reflects additive‐specific interfacial interactions rather than a universal stability criterion. Considering the differences in role of glycerol, we can predict that glycerol will have an exact opposite reorienting ability of Zn deposition than PVP which leads to better battery performance. Interestingly, that is what we observed in Figure 6(E), where the relative intensity ratio of (002) and (100) planes were the highest for 10% G and it drastically decreased with increased concentrations. This result alings with the proposed hypothesis that upto 10% G solvation energetics dominate and beyond that mass trasnport limitations lead to unstable zinc plating with lower (002), accelerating HER. Which also explains why 10% G gave the best anode stability while in case of PVP, 50% concentration was the best. It is important to realize that for 0% G, the relative intensity ratio was 1.25, which is much lower than 10% G and almost similar with 30% or 50% G ratios. This trend aligns with trend of CE values shown in Figure 4(C), with an increase in overall performance for 10% G due to optimal solvent reorganization kinetics but beyond that the CE values decreased with increasing additive concentrations. These results clarify the proposed hypothesis that up to the optimal additive concentration, a synergistic interplay between nucleation and growth kinetics drives uniform zinc deposition with preferential crystallographic orientations that intrinsically suppresses HER kinetics.

**FIGURE 7:**
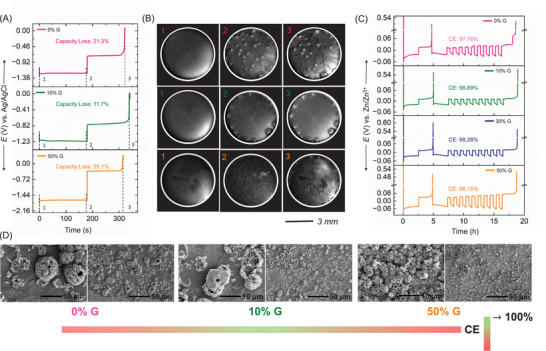
(A) Potentiometry at 20 mA/cm^2^ current density and 1 mAh/cm^2^ capacity, for different glycerol content. A Cu foil with a 3 mm radius was used as the working electrode, Zn strip served as the counter electrode and Ag/AgCl in 1 M KCl served as reference electrode. Points labeled 1–3 indicate positions where optical microscopy images were captured. (B) In situ optical microscopy images of Zn electrodeposition and stripping in various electrolytes, taken at the corresponding marked points in the potentiometry curve. (C) Aurbach protocol for evaluating Zn stripping/plating Coulombic efficiency (CE) with varying glycerol content in Cu|Zn asymmetric cells. (D) Scanning electron microscopy images taken after stripping, showing dead Zn and/or corrosion products.

Figure 7 presents a comprehensive investigation of dead Zn and corrosion product formation in electrolytes with varying glycerol content, using potentiometric analysis and in situ optical microscopy. A three‐electrode configuration was employed, with a 3 mm‐radius Cu foil as the working electrode, Ag/AgCl in 1 M KCl as the reference electrode, and a Zn strip as the counter electrode. A cathodic current density of 20 mA cm^−^^2^ was applied for 3 min to deposit Zn onto the Cu surface, followed by an anodic current of 20 mA cm^−^^2^ to strip the deposited Zn. During this process, real‐time imaging of the electrode surface was performed using in situ optical microscopy. Figure 7(A) displays the corresponding voltage–time profiles, while Figure 7(B) shows optical microscopy images at selected time points. Capacity loss in different electrolytes was calculated using Equation (6):

(6) $$C_{loss}=\frac{t_{c}-t_{d}}{t_{c}}\times Q_{load}$$

where *t*_c_ and *t*_d_ represent the charge and discharge times, respectively, and *Q*_load_ is the capacity of 1 mAh cm^−^^2^. The calculated capacity losses, shown as percentages in Figure 7(A), revealed that the lowest loss (11.2%) occurred at 10% G, while higher losses were observed at 0% (21.3%) and 50% G (25.1%). The elevated capacity loss at 50% G is attributed to reduced ionic mobility and increased nucleation and stripping overpotentials, indicative of greater formation of dead Zn and corrosion products. These results suggest that an optimal glycerol concentration minimizes irreversible Zn loss. The corresponding optical images (Figure 7(B)) further corroborate these findings. At point 1, corresponding to the onset of Zn deposition, bubble formation, associated with HER, was most pronounced in the 50% G electrolyte, consistent with the ECMS data in Figure 6(D). At 10% G, significantly fewer bubbles were observed, suggesting suppressed HER. Point 2, taken just before Zn stripping, showed more trapped gas bubbles in 0% and 50% G compared to 10% G. During the charging process, continuous and vigorous hydrogen evolution was visually evident in 50% G, whereas it was significantly reduced in 10% and 0% G (as seen in Movies S1–S3). Point 3, corresponding to the post‐stripping state, captured residual Zn and/or corrosion products on the Cu surface. Notably, the 10% G condition exhibited the least residue, indicating improved reversibility. Quantitative assessment of irreversible zinc accumulation (dead Zn and corrosion products) is provided separately by electrochemical quartz crystal microbalance (EQCM) analysis over multiple cycles (Figure S24). The stripped‐to‐plated mass ratio obtained from EQCM measurements quantifies mass reversibility during cycling. A ratio approaching unity indicates efficient stripping of deposited Zn^0^, whereas lower ratios signify irreversible mass accumulation arising from dead zinc formation and corrosion products. The maximum mass reversibility is observed at the optimal glycerol concentration, consistent with enhanced CE and suppressed parasitic reactions.

These trends were further validated by the Aurbach test on asymmetric coin cells with different glycerol concentrations, with the experimental protocol described in the SI [10, 67, 68, 69]. As shown in Figure 7(C), 10% G yielded the highest CE, while higher glycerol concentrations led to a decline in CE, consistent with increased irreversible Zn loss. Figure 7(D) presents SEM images of the Cu electrode post‐stripping. At 0% G, sparse flake‐like Zn residues were observed. In contrast, 10% G resulted in more compact and finer residue with minimal surface coverage, while 50% G showed extensive coverage with large, porous, and flaky structures. These morphological observations, together with the potentiometric data, EQCM data, optical imaging, and Aurbach analysis, collectively confirm that the optimal additive concentration minimizes dead Zn formation and corrosion products. The schematic in Figure 7(D) summarizes this finding, highlighting that an optimal balance between charge transfer, nucleation and corrosion kinetics, mediated by solvation and reorganization dynamics, is achieved at the optimal glycerol concentration. Deviation from this optimum point, either by increasing or decreasing the additive content, disrupts this balance and leads to enhanced side reactions and reduced battery performance. Note that, while “controlled” decrease of zinc deposition kinetics enhances anode stability, its translation to full‐cell performance requires balancing anode protection with acceptable polarization at the cathode. The extension of this optimal kinetic window to cathode‐specific chemistries represents an important direction for future investigation.

## 3 Conclusions

Our study provides a complementary view to the prevailing notion that continuously lowering *i*_0,Dep_ enhances zinc anode performance and instead reveals that electrolyte additives exhibit an optimal concentration governed by a balance between charge transfer kinetics, solvent reorganization dynamics, mass transport, and side reaction suppression. While slowing *i*_0,Dep_ initially improves coulombic efficiency and zinc morphology, excessive suppression of charge transfer kinetics leads to increased HER and dendritic growth, ultimately degrading performance. By employing temperature‐dependent studies and model additive experiments with glycerol (G) and polyvinylpyrrolidone (PVP), we demonstrate that this trend is dictated by a synergy of multiple competing processes. Our findings highlight the inadequacy of traditional models such as the Sand's model and classical nucleation theory in fully describing electrolyte additive interactions. Furthermore, our study shows that the optimal additive concentration minimizes dead Zn and corrosion product formation, leading to the highest reversibility and coulombic efficiency. Deviating from this balance, either with lower or higher additive content, accelerates irreversible Zn loss, competetive HER, and leads to overall performance degradation. By introducing a refined mechanistic framework, we provide critical insights into the design of next‐generation electrolytes that balance charge transfer of zinc plating, corrosion as well as mass transport efficiency to achieve anode stability, offering a more comprehensive strategy for improving the longevity and reliability of AZMBs.

## Author Contributions

M.A.F., A.R. and J.E.D conceived the idea. M.A.F., T.L. and A.R. did all the electrochemical and coin cell experiments and data analyses. J.H.N. and S.P. did the SEM and XRD characterization. A.B. and B.M.T did the ECMS experiment. M.A.F., T.L. and A.R. wrote the paper and all authors edited it. J.E.D. supervised all aspects of the work. All authors have agreed to the final version of the manuscript.

## Conflicts of Interest

There are no conflicts of interest to declare.

## Supporting information




**Supporting File 1**: anie71489‐sup‐0001‐SuppMat.docx.

**Supporting File 2**: anie71489‐sup‐0002‐MovieS1.mp4.

**Supporting File 3**: anie71489‐sup‐0003‐MovieS2.mp4.

**Supporting File 4**: anie71489‐sup‐0004‐MovieS3.mp4.

## Data Availability

The data that support the findings of this study are available from the corresponding author upon reasonable request.
