# Supplementary material for: Slowing Down Zinc Electrodeposition Kinetics Can Maximize and Compromise Anode Stability: How Slow Is Too Slow?
Source: Angew Chem Int Ed Engl. 2026 Mar 10;65(17):e1181269. doi: 10.1002/anie.1181269 (PMC13098480; doi:10.1002/anie.1181269)
Supplement: Supplementary file 1 — Supporting File 1: anie71489‐sup‐0001‐SuppMat.docx. [file ANIE-65-e1181269-s004.docx]

**Supporting**

**Information**

**Slowing Down Zinc Electrodeposition Kinetics Can Maximize and Compromise Anode Stability: How Slow is Too Slow?**

Md. Arif Faisal^a,£^, Taizhe Liu^a,£^, Ashutosh Rana^a,£^, James H. Nguyen^a^, Saptarshi Paul^a^, Ashutosh Bhadouria^a,c^, Brian M. Tackett^a,c^ and Jeffrey E. Dick^a,b^*

^a^ Department of Chemistry, Purdue University, West Lafayette, IN, 47907, USA

^b^ Elmore Family School of Electrical and Computer Engineering, Purdue University, West Lafayette, IN, 47907, USA

^c^ Davidson School of Chemical Engineering, Purdue University, West Lafayette, IN, 47907, USA

**Corresponding Author:**

Jeffrey E. Dick ([jdick@purdue.edu](mailto:jdick@purdue.edu))

**Experimental Section**

1. **Materials**

Reagent-grade Zinc Chloride (ZnCl_2_), Hexaammineruthenium (III) chloride, Reagent-grade Potassium Chloride (KCl), Glycerol (≥99.5% purity) and Polyvinylpyrrolidone (PVP) were purchased from Sigma-Aldrich. Zinc foil and Copper foil (99.99% purity) were sourced from MTI Supplies. 25 µm Tungsten and Copper metal wires were obtained from Goodfellow. All chemicals were used as received without further purification. Deionized (DI) water for preparing aqueous electrolytes was obtained from a Milli-Q ultrapure water system.

1. **Preparation of the Electrolyte**

The electrolytes were prepared by dissolving 1 mol/L (M) ZnCl_2_ in deionized (DI) water at room temperature (25 °C). Subsequently, varying amounts of glycerol (G) and PVP powders were added to the 1 M ZnCl₂ solution, resulting in 5%, 10%, 20%, 30%, 40%, and 50% volume ratios of glycerol to ZnCl₂ and 10%, 30%, and 50% weight ratios of PVP to ZnCl₂.

1. **Method of Testing the W Ultramicroelectrode**

A two-electrode setup is used for CV measurements when utilizing a homemade W UME as the working electrode. This is because the small current passed through the system during measurements minimizes potential drop at the working electrode, allowing its potential to be reliably referenced against the stable potential of the Ag/AgCl (1 M KCl) reference/counter electrode. All electrochemical measurements were performed with 1 M ZnCl_2_ as the supporting electrolyte. Before initiating zinc electrodeposition on the Tungsten UME, a cyclic voltammogram was recorded using an outer-sphere redox molecule, hexaammineruthenium(III) chloride (RHT), to confirm the presence of steady-state currents, characteristic of UMEs. **Fig. S26 s**hows a sigmoid-shaped cyclic voltammogram obtained with the same experimental setup, using 5 mM RHT as the electrolyte. The observed low capacitance and steady-state currents validate the proper fabrication of the UME.^52^

1. **Characterization**

Zinc foils were converted into discs and used as Zn electrodes for symmetric cell, whereas Copper discs were used as Cu electrodes for asymmetric cell. The zinc is deposited on the Copper foil for x-ray diffraction measurement (XRD). The SEM was done using field emission scanning electron microscopy (FESEM, JEOL JSM-7500F). Phase structure exploration was performed by XRD (Bruker D8 advanced) with Cu Kα radiation over the 0° to 45° 2 theta range. Zn nuclear magnetic resonance (^67^Zn NMR) were carried out by Bruker NMR (500 MHz and 300 MHz respectively).

1. **Electrochemical Measurements**

All electrochemical measurements were conducted using a CHI6284E electrochemical workstation. As detailed in the main text, a two-electrode configuration was employed for the CV measurements. Rotating disk electrode (RDE) was performed by CHI potentiostat in a three-electrode setup, where a 3 mm diameter of glassy carbon macroelectrode (Pine Research) was used as the rotating working electrode, Zn foil as a counter electrode and an Ag/AgCl in 1 M KCl as the reference electrode.

1. **Equations for Tafel analysis**^1^

**Cathodic Reaction:** $Log\left| i \right|=Log\left( i_{0} \right)-\left( \frac{\alpha F}{2.3RT} \right)\eta$

**Anodic Reaction:** $Log\left| i \right|=Log\left( i_{0} \right)+\left( \frac{(1-\alpha)F}{2.3RT} \right)\eta.$

Where $i$is current, $i_{0}$ is exchange current, $\eta$is overpotential, $\alpha$is the transfer coefficient, $F$ is Faraday’s constant, *T* is the temperature in Kelvins, $R$is universal gas constant.

**7. Details of Fast Scan Cyclic Voltammograms and Measurement of Diffusion Coefficient**

In **Fig. 4(b)**, the cyclic voltammogram of zinc electrodeposition on a tungsten UME (radius ~12.5 μm) have been represented, where a two-electrode setup with an Ag/AgCl in 1 M KCl as the counter/ reference electrode and the UME as the working electrode was utilized. The voltammetry shown at the top of **Fig. 4(b)** was conducted between 0.0 V and -1.6 V at a scan rate of 0.05 V/s to study Zn electrodeposition. During the forward scan, from 0.0 V to -1.6 V, Zn deposition began at approximately -1.1 V, with a diffusion-limited peak observed near -1.4 V before reaching the switching potential. As the scan reversed at -1.6 V, a characteristic nucleation loop appeared, typical of metal electrodeposition. Notably, zinc deposition persisted even after the voltage sweep direction changed, until a crossover from negative to positive current was detected. At the crossover voltage, the current began to rise, indicating the stripping of deposited Zn from the electrode, which culminated in a current peak known as the peak stripping current. Similarly, at the bottom of **Fig. 4(b)**, the zinc electrodeposition voltammetry at a faster scan rate (60 V/s) is represented, where mass-transfer effects are negligible as shown in our previous works.^50,51^

Furthermore, the slow scan rate voltammetry showed that with increasing additive concentration, the stripping peak current decreased with increasing additive concentration. This is because the charge of deposition (*Q*_Dep_) decreased with increasing glycerol concentration in the electrolyte (see **Fig. S25** and **Table S1**), which caused reduced stripping in response. The shoulder peaks observed after the stripping peak in the slow scan voltammogram are indicative of the stripping of zinc from the bare electrode. According to our previous findings, the non-overlaying nature of the kinetic region (10% of the peak current region) in **Fig. S6**, are indicative of the “slow scan regime” where the mass-transfer dominates and convolutes with the charge transfer kinetics leading to a growth-controlled process.^50,51^ On the contrary, the overlaying nature of the kinetic region in **Fig. S5** suggests that they are in “fast-scan regime”, where the deposition is charge-transfer controlled. Clearly, there were very little change in the voltammetry with the addition of 10% G in both slow and fast scan rates. However, drastic changes were observed for the increased concentrations. For higher additive concentrations, the diffusion of the Zn^2+^ ion was harder which in turn may cause sluggish charge transfer kinetics. The diffusion coefficient in the presence and absence of glycerol in 1 M ZnCl_2_ is represented in **Fig. 4(d)**, using the peak observed during the forward sweep of the FSCVs. The equation of peak current as a function of scan rate for irreversible redox reaction,^51^

$j_{p}=(2.99\times{10}^{5})n^{\frac{3}{2}} C_{Zn^{2+}}\alpha^{\frac{1}{2}}{D_{Zn^{2+}}^{\frac{1}{2}} \nu}^{\frac{1}{2}}$……………………………….(1)

was used to determine the diffusion coefficient using the slope of the fit between $j_{p}$ and $\nu^{0.5}$, as shown in **Fig. S7**. Where, $j_{p}$​ is the peak current density (peak current divided by geometric area), $n$ is the number of electrons transferred, $C_{Zn^{2+}}$ is the concentration of zinc ions, $\alpha$ is the transfer coefficient, $D_{Zn^{2+}}$is the diffusion coefficient of Zn^2+^ and $\nu$ is the scan rate. With increased glycerol concentration, the diffusion coefficient decreased drastically which suggests the increased viscosity of the electrolyte due to added additive.

Also, the exchange currents were calculated by Tafel analyses from the FSCVs (**Fig. S5**) and represented along with the CE values in **Fig. 4(c)**. The CE values were obtained from the FSCVs of different glycerol concentrations and determined by following equation 5.

$Coulombic efficiency=\frac{Q_{stripping}}{Q_{deposition}}\times100\%$..................................(2)

Note that we used the FSCVs for the CE measurements to eliminate any consequences from mass transfer effects on deposition or stripping of zinc. Therefore, the exchange current values are denoting to the true charge transfer of zinc deposition which is the *i*_0,Dep_.

**8. Details of Electrochemical Mass Spectrometry (ECMS)**

In the following section, we present electrochemical mass spectrometry (ECMS) as a powerful technique for the real-time quantification of hydrogen evolution reaction (HER) activity during zinc electrodeposition. For our measurements, helium gas with a research-grade purity of 6N (sourced from A-OX Welding) was used as the carrier gas. This helium stream was regulated through an internal mass flow controller, which not only maintained a consistent pressure differential but also enabled the efficient transport of evolved gaseous species to the mass spectrometer (MS) inlet for analysis.

The electrochemical cell employed a three-electrode configuration. The working electrode consisted of a copper (Cu) disk with an active area of approximately 0.196 cm². An Ag/AgCl reference electrode immersed in 3.4 M KCl solution (supplied by eDAQ) provided a stable reference potential, critical for accurate thermodynamic assessments. A platinum (Pt) wire was used as the counter electrode. This choice—over a zinc counter electrode—was intentional, as the study focuses exclusively on electrochemical processes occurring at the copper working electrode, and thus any reaction at the counter is not of direct interest.

All electrodes were interfaced with a Biologic SP-300 potentiostat, controlled through EC-Lab software to carry out the electrochemical experiments. The ECMS system was operated using Zilien software developed by Spectro Inlets, which facilitated the control of valves, MS operation, and real-time data acquisition. For subsequent data analysis, we utilized the ixdat Python package, specifically designed for processing ECMS datasets.

**9. Aurbach Protocol for Coin Cell Performance**^68^

Substrate-related factors such as lattice mismatch, alloy formation, and interphase interactions can impact Coulombic efficiency (CE) measurements in various coin cell configurations.^10,69^ To isolate the effect of different zinc electrolytes and mitigate these substrate influences, we employed the galvanostatic method described by Adams et al. and Xu et al., as illustrated in **Fig. 7(c)**.^69,70^ Specifically, to ensure consistency across all concentrations, an initial conditioning cycle was performed, during which 5 mAh cm⁻² of Zn was deposited onto and removed from the Cu working electrode at a current density of 2 mA/cm², with a cutoff voltage of +0.5 V. Following this step, an additional 5 mAh cm⁻² of Zn was plated onto the Cu (acting as a reservoir), providing a controlled and finite Zn source for accurate CE assessment (Q_r_) at the same current density. The cycling protocol then involved repeated deposition and stripping of a fixed 1 mAh cm⁻² (Q_c_) for nine cycles, followed by complete Zn removal to +0.5 V versus Zn/Zn²⁺ to ensure all extractable Zn, including the initial reservoir (Q_s_), was stripped. The CE was determined using Equation 1.

$CE=\frac{{9Q}_{c}+Q_{s}}{{9Q}_{c}+Q_{r}}$………………………………………………(1)


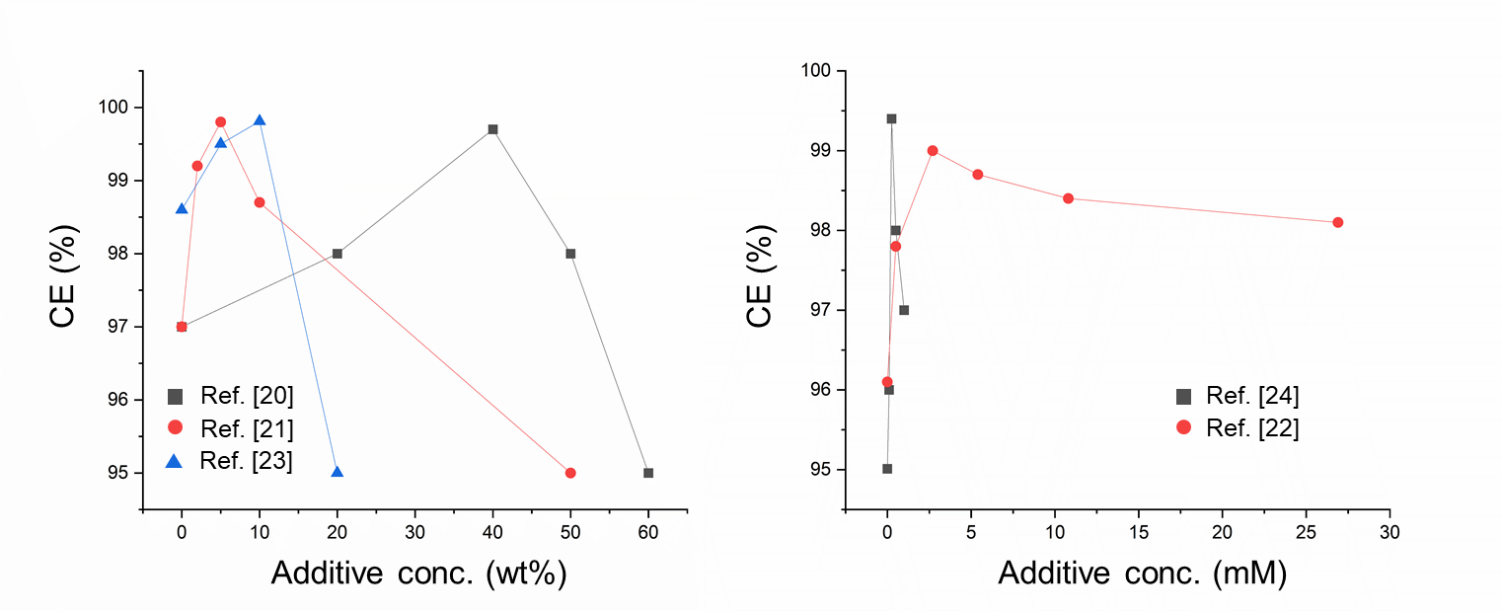


**Fig S1.** Coulombic efficiency vs. additive concentration plot for different additives, obtained from the literature, showing the optimal concentration of each additive. The references are listed in the main file and the reference numbers are also indicative of the main file’s reference list.


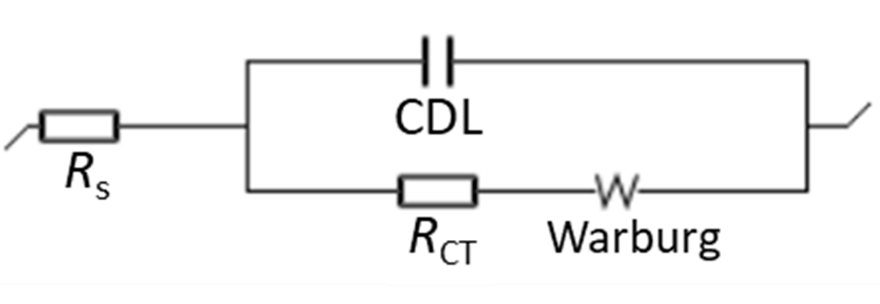


**Fig S2.** Equivalent circuit model used to fit electrochemical impedance spectroscopy (EIS) data. The circuit consists of the solution resistance (*R*_s_​) in series with a parallel combination of the double-layer capacitance (CDL​) and the charge-transfer resistance (*R*_CT_​), followed by a Warburg diffusion element (W) to account for mass-transport effects during zinc deposition and stripping.


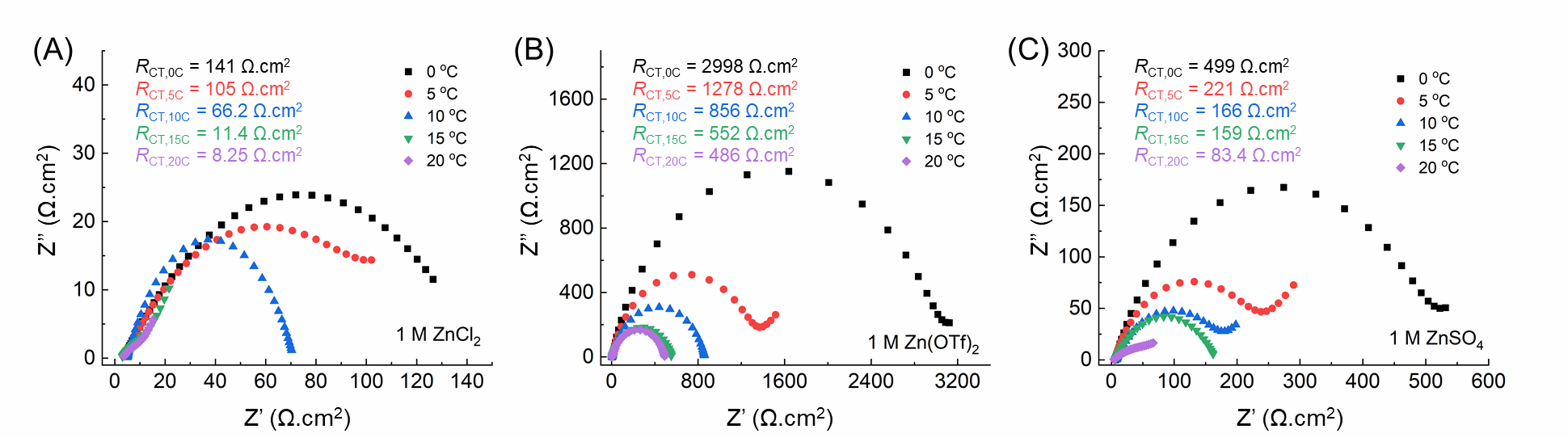


**Fig S3.** Temperature-dependent electrochemical impedance spectroscopy (EIS) measurements of different zinc electrolytes. A three-electrode setup was used where a zinc macroelectrode served as working electrode, Pt as counter electrode and Ag/AgCl in 1 M KCl as reference electrode. (a) Nyquist plot at different temperatures for 1 M ZnCl_2_, (b) ZnOTf_2_ and (c) ZnSO_4_ electrolytes. The charge transfer resistance (*R*_ct_) values were extracted by fitting the circuit shown in Fig. S2.


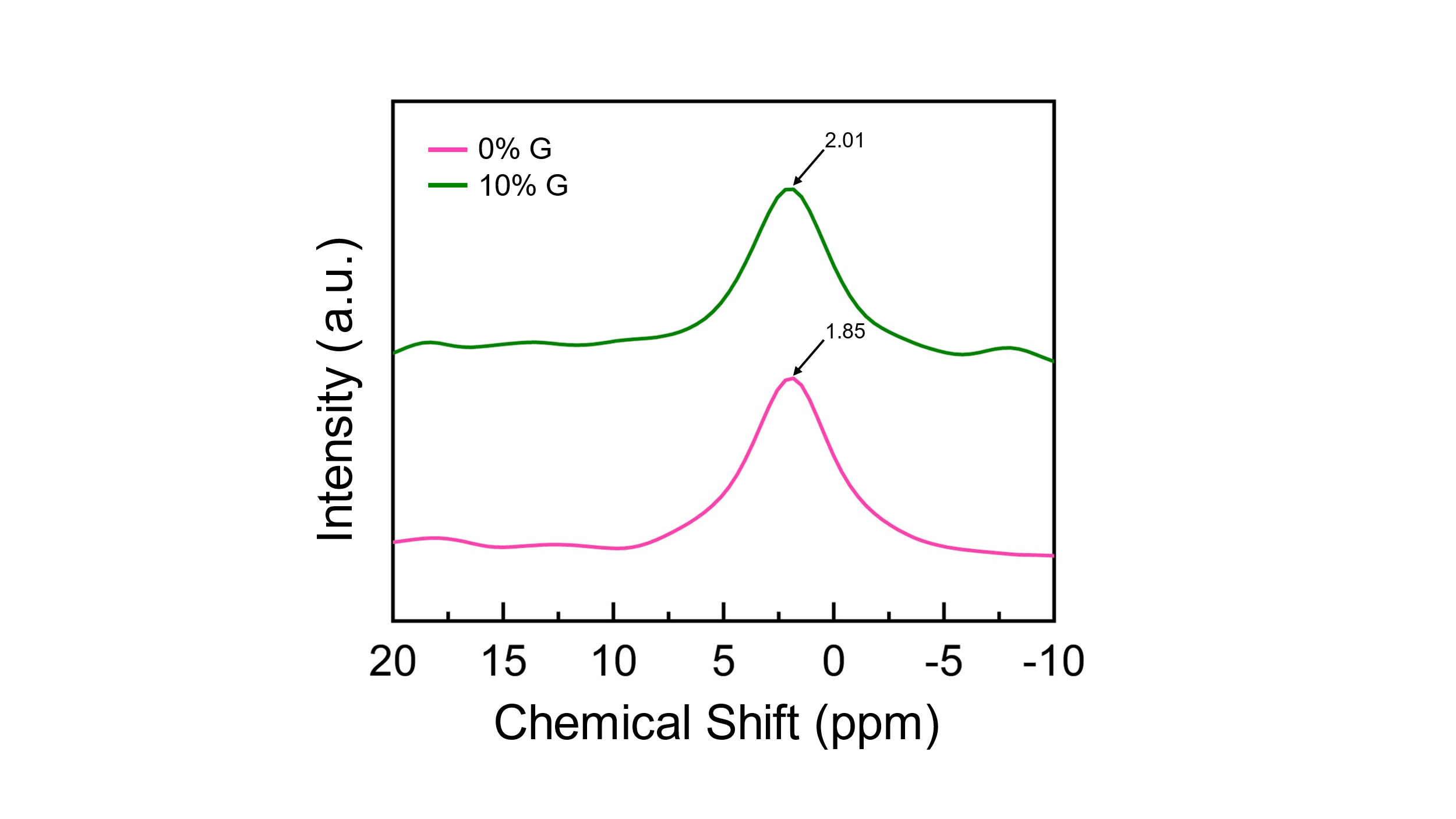


**Fig S4.** ⁶⁷Zn NMR for 1 M ZnCl₂ solution containing 0% and 10% glycerol additive.


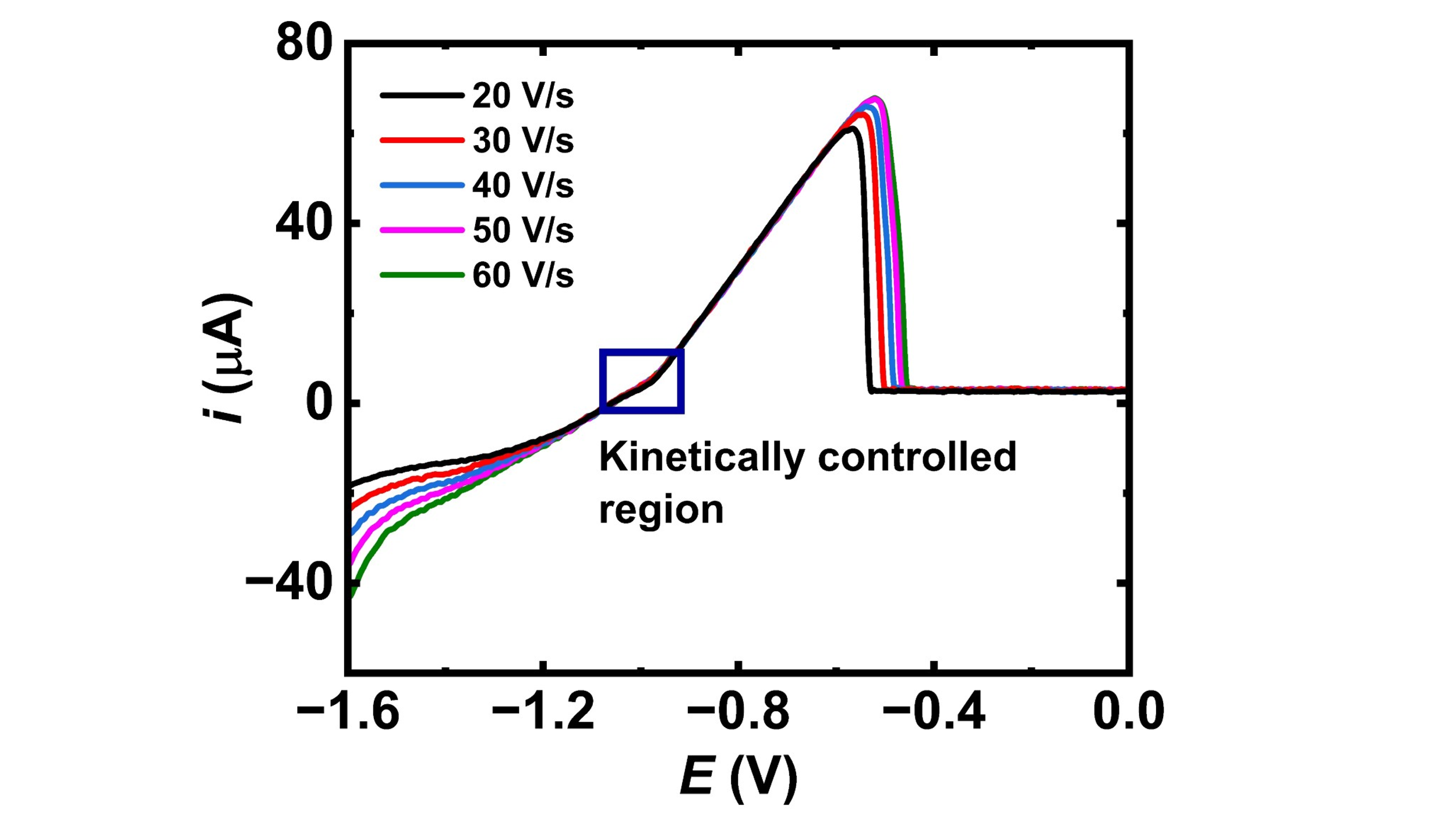


**Fig S5.** The overlap of backward sweep at kinetically controlled region between 20 Vs^-1^ to 60 Vs^-1^ (fast scan regime) performed at the two-electrode setup mentioned above.


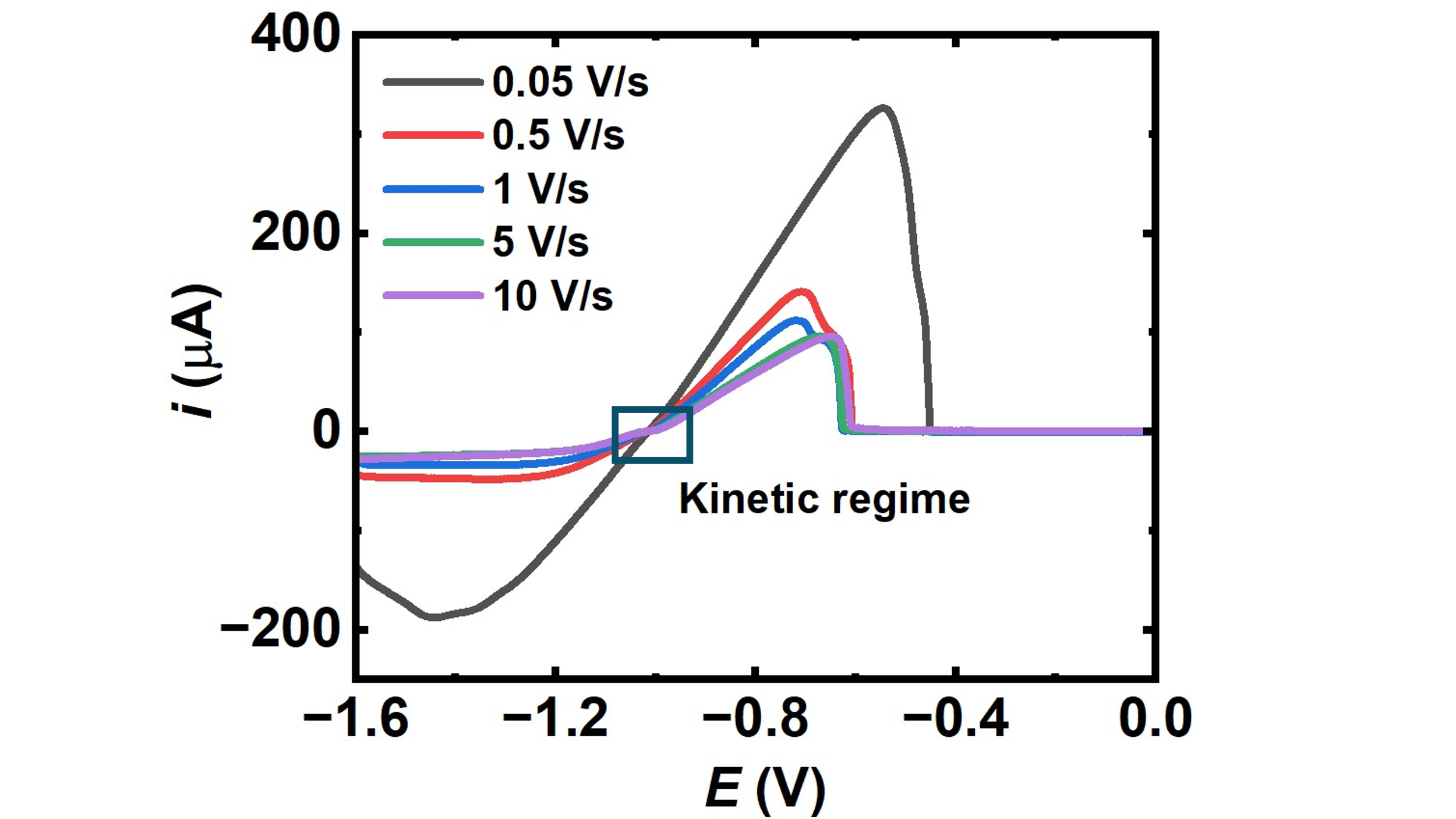


**Fig S6.** The overlap of backward sweep at scan rate between 0.05 Vs^-1^ to 10 Vs^-1^ (slow scan regime) characterizes non-overlaid kinetic region, performed at the two-electrode setup mentioned above.


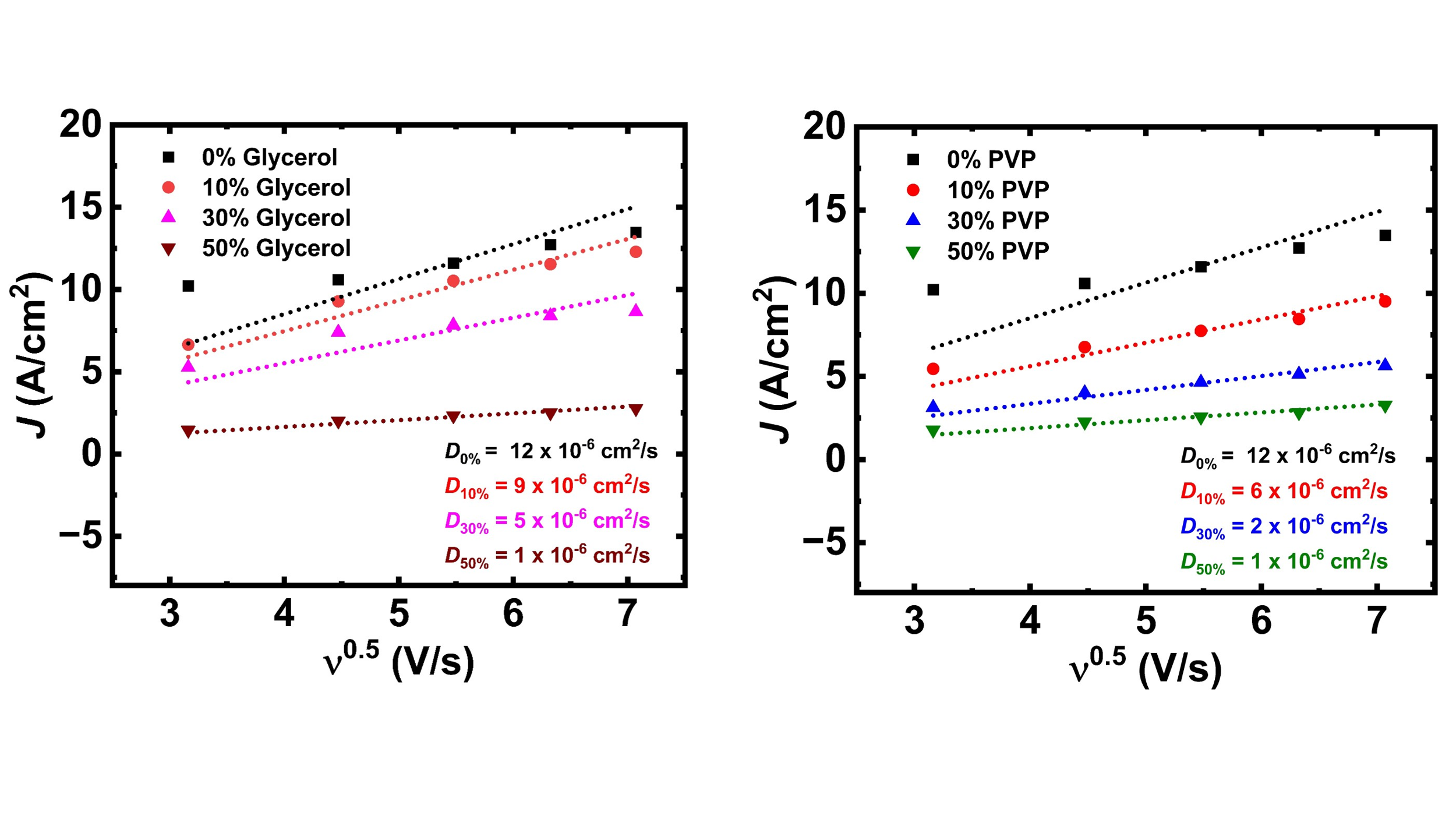


**Fig S7.** Diffusion coefficient ($D_{Zn^{2+}}$) of Zn^2+^ in presence and absence of different concentrations of glycerol and PVP in 1 M ZnCl_2_.


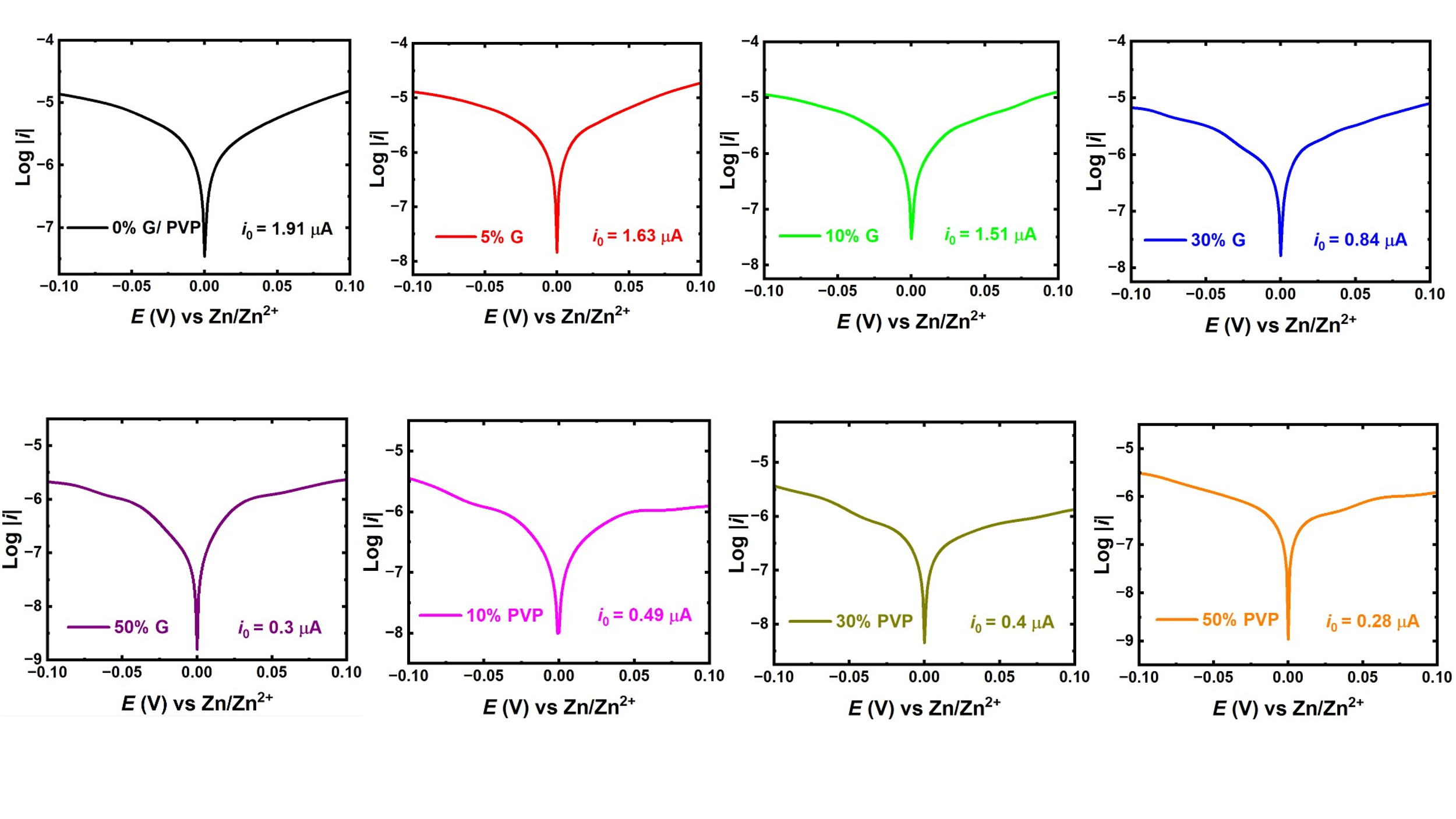


**Fig S8.** Tafel plots for 0%, 5%, 10%, 30%, 50% Glycerol and 0%, 10%, 30%, 50% PVP at a scan rate of 60 Vs^-1^ using the methodology detailed in the main file.


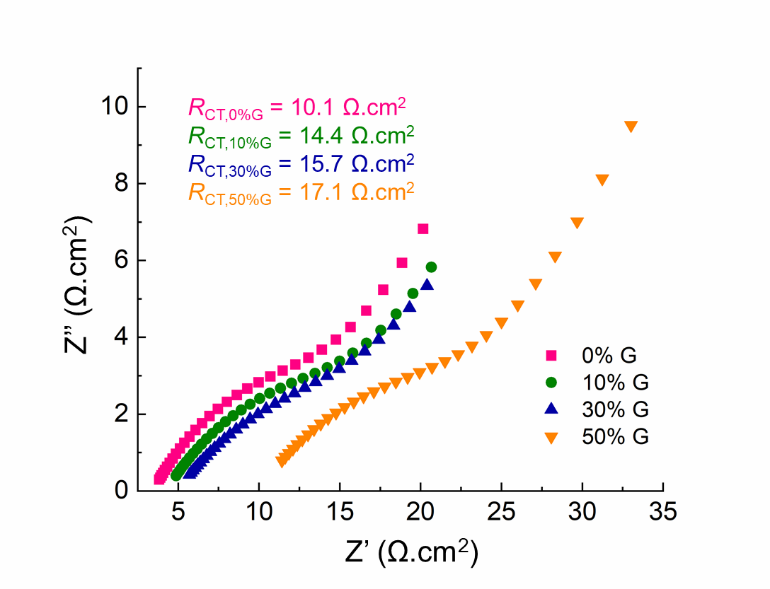


**Figure S9.** Nyquist plots obtained from electrochemical impedance spectroscopy (EIS) measurements of Zn electrodes in electrolytes containing different glycerol concentrations (0%, 10%, 30%, and 50% G). The charge transfer resistance (*R*_ct_) values were extracted by fitting the circuit shown in Fig. S2.


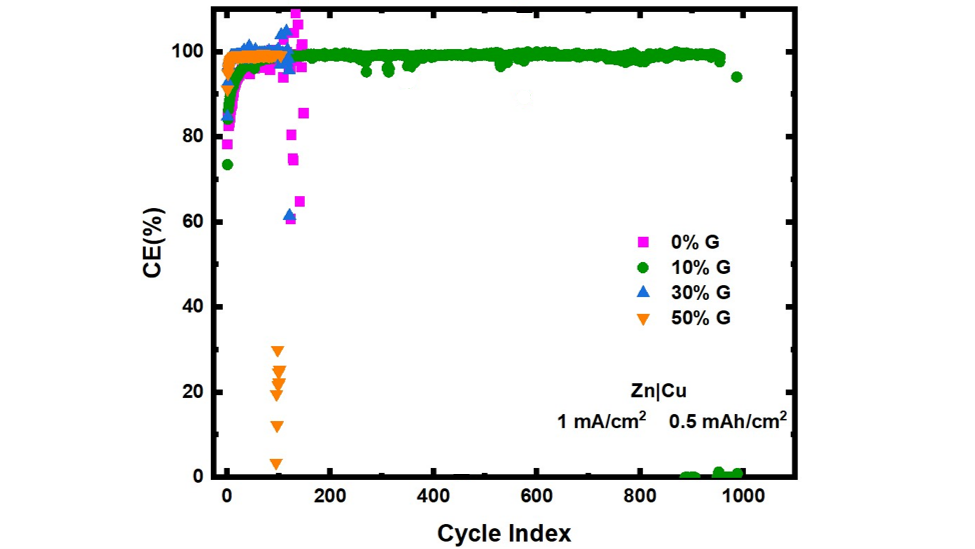


**Fig S10.** Cu|Zn asymmetric cell cycling at a current density of 1 mA/cm² and a capacity of 0.5 mAh/cm² in 1 M ZnCl₂ under the varying concentrations of Glycerol.


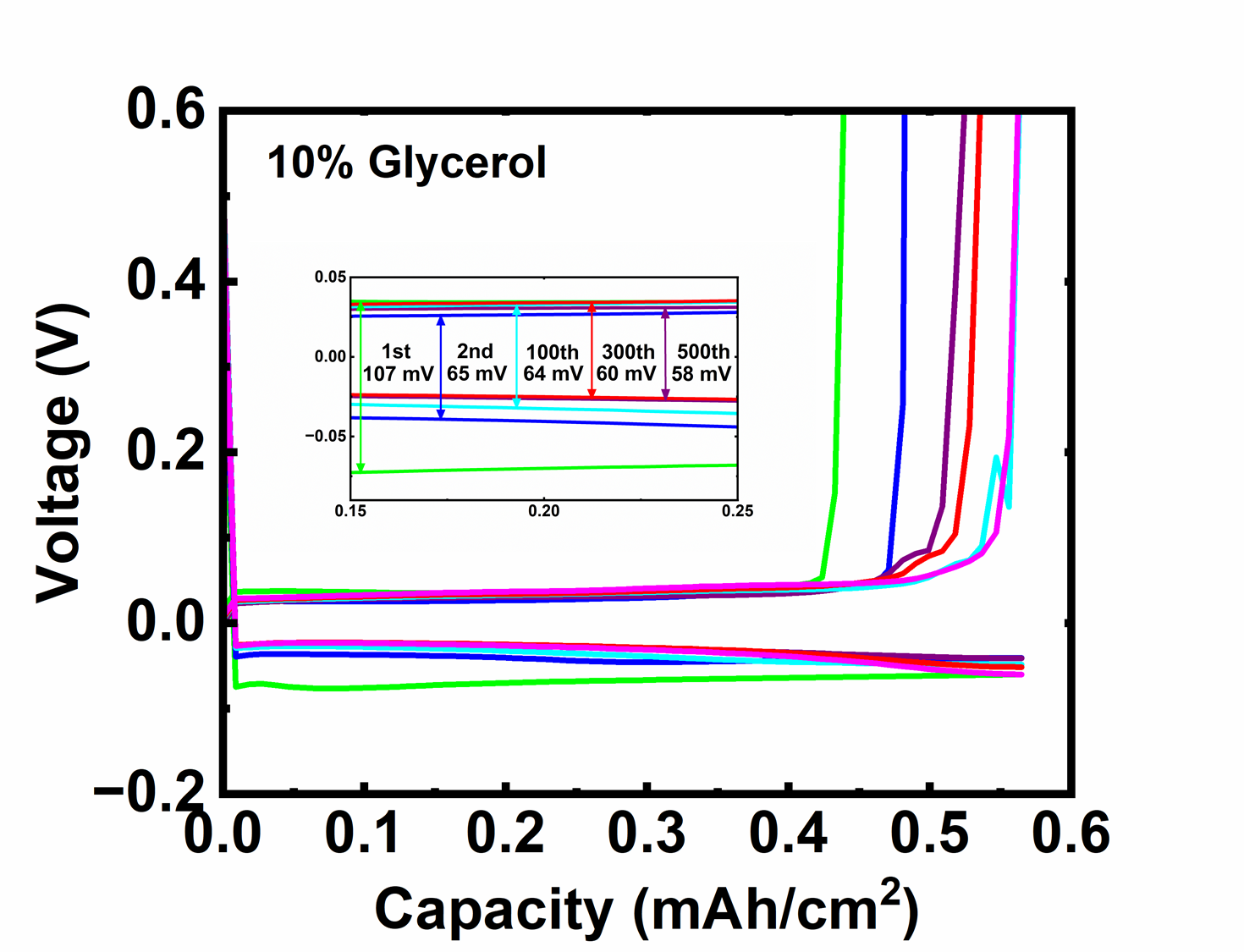


**Fig S11.** Stable charging/discharging profile of the 10% G additive in Cu|Zn asymmetric coin cell.


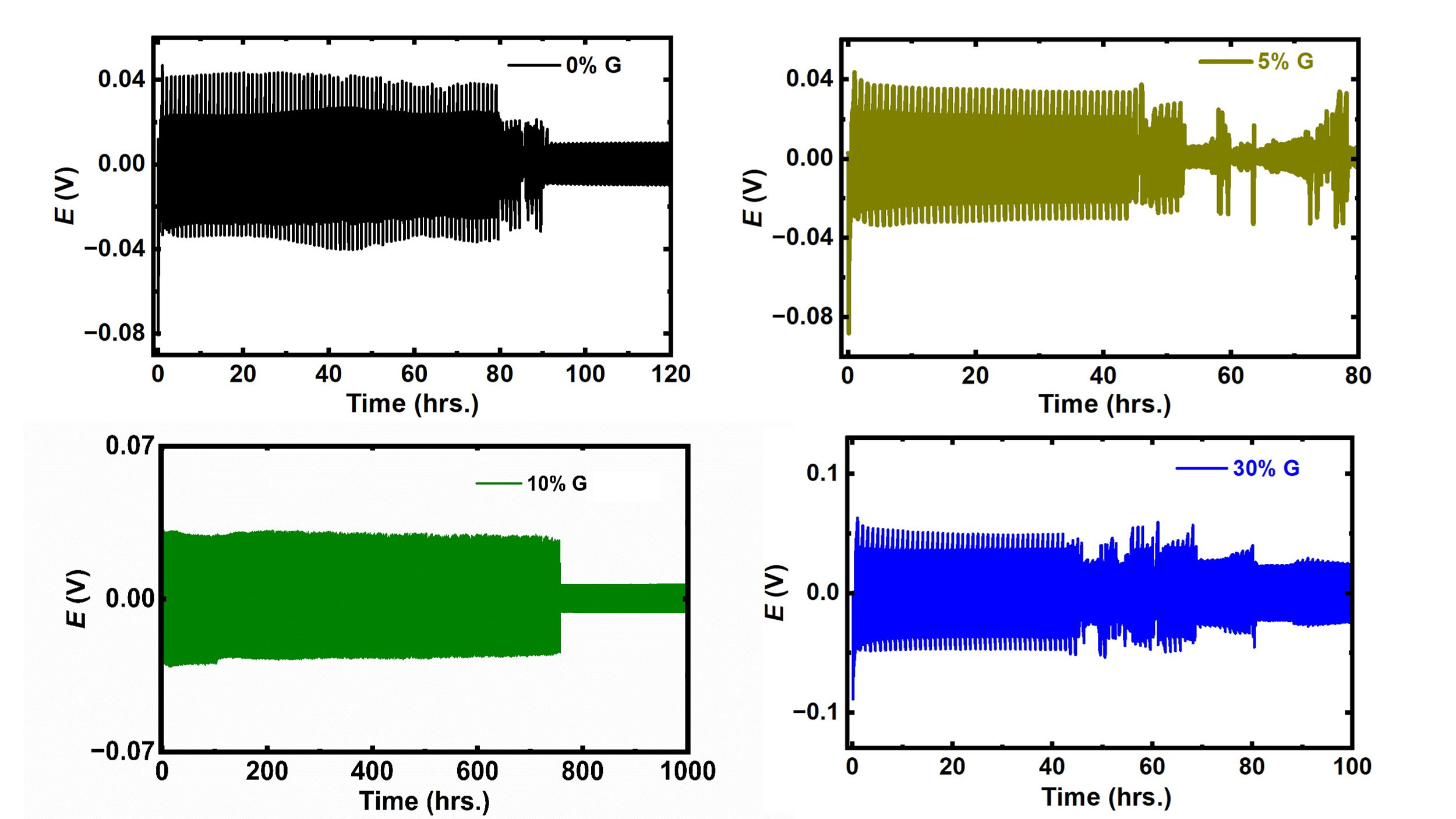

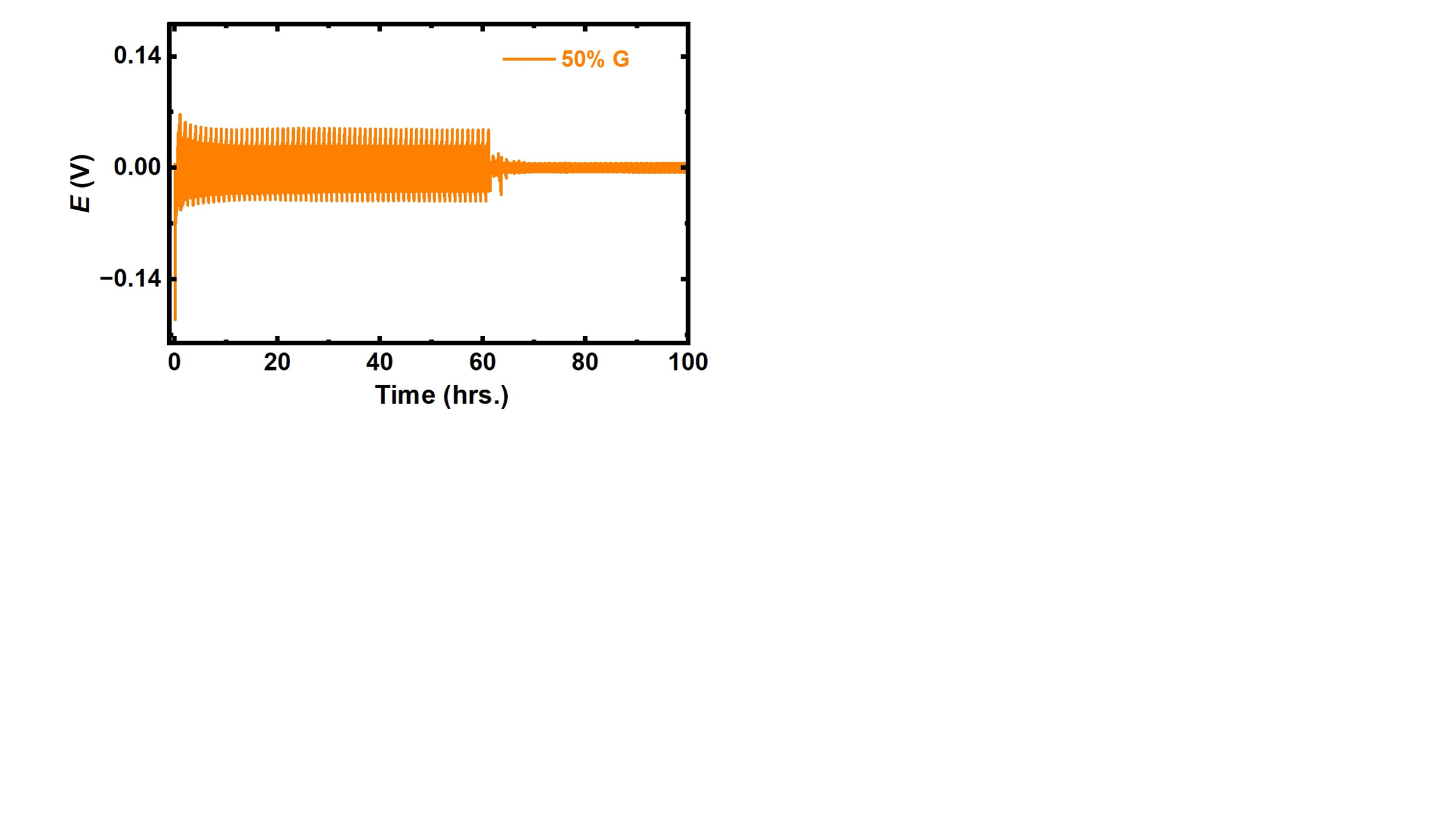


**Fig S12.** Zn|Zn symmetric cell cycling at a current density of 1 mA/cm² and a capacity of 0.5 mAh/cm² in 1 M ZnCl₂ under the varying concentrations of Glycerol.


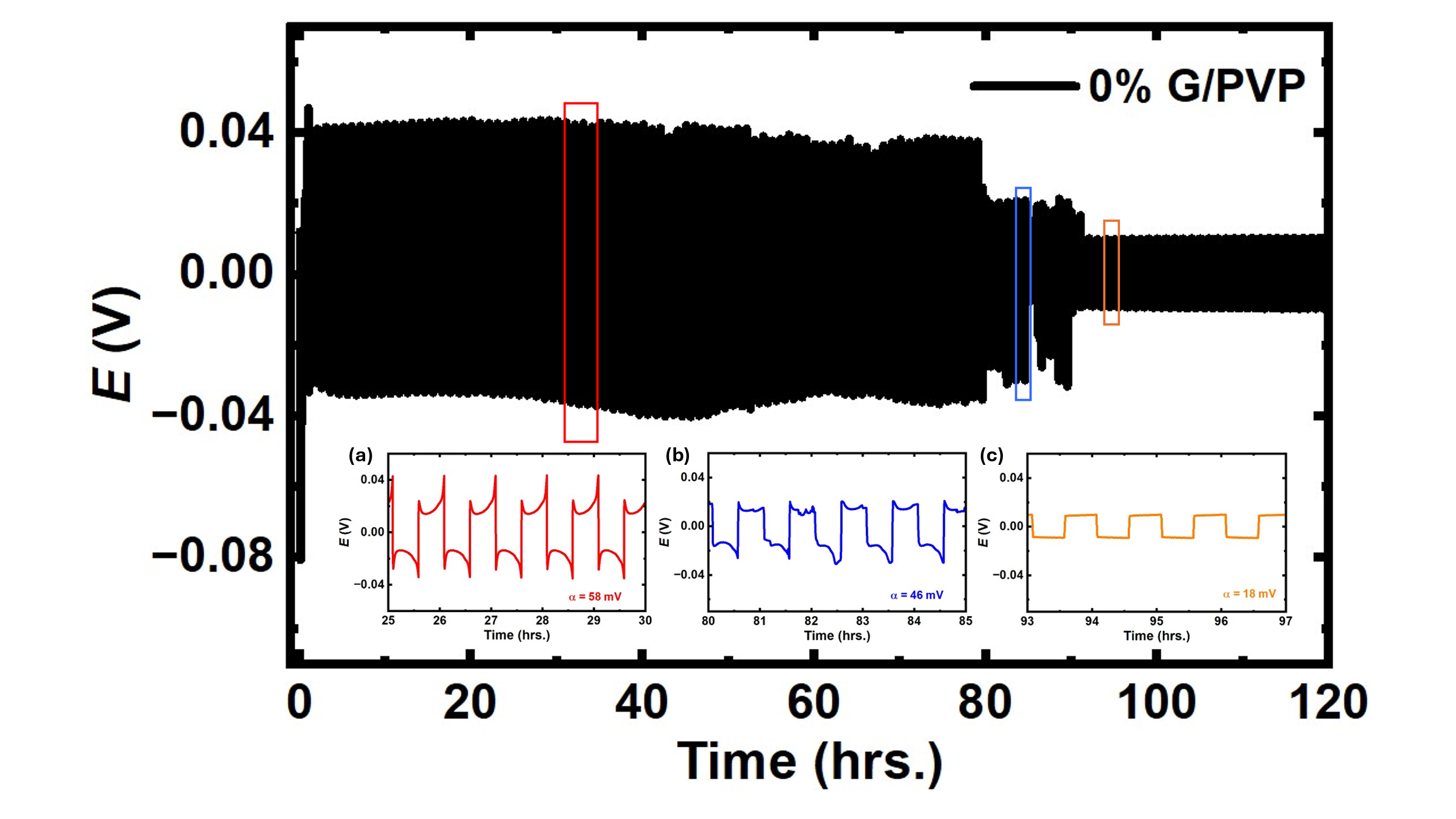


**Fig S13.** Zn|Zn symmetric cell cycling of 1 M ZnCl₂ at a current density of 1 mA/cm² and a capacity of 0.5 mAh/cm². Close up figures (a), (b), and (c) shows the fluctuant polarization voltages with cycles.


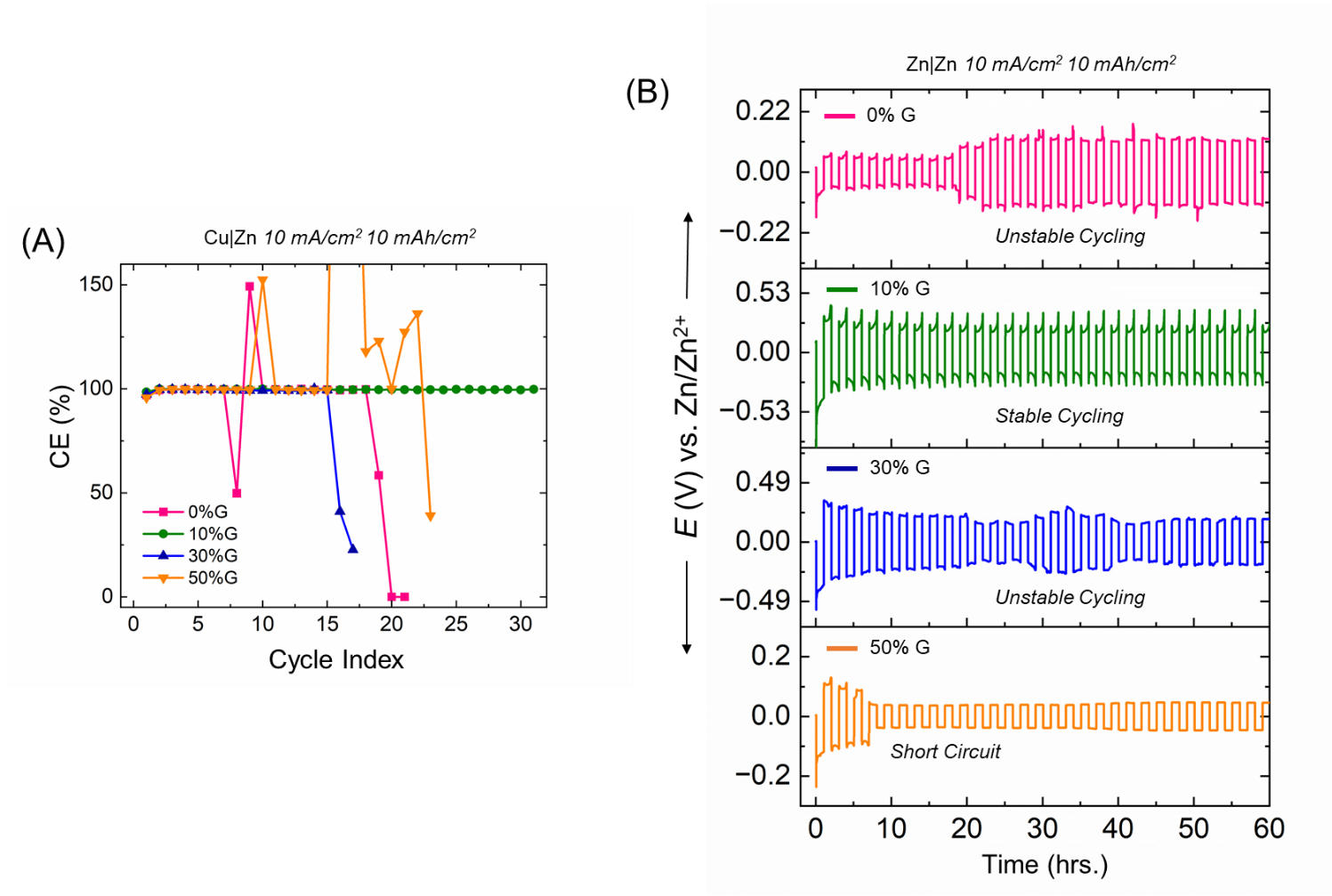


**Figure S14.** Electrochemical performance of coin cells containing different glycerol concentrations under high current density (10 mA cm⁻²) and high areal capacity (10 mAh cm⁻²). (a) Coulombic efficiency (CE) evolution in asymmetric Cu‖Zn cells. (b) Galvanostatic cycling profiles of symmetric Zn‖Zn cells for 0%, 10%, 30%, and 50% glycerol electrolytes. The optimal glycerol concentration exhibits the most stable cycling behavior, whereas glycerol-free and excessively glycerol-rich electrolytes show unstable cycling or premature short-circuiting, confirming that the optimal kinetic window persists under aggressive operating conditions.


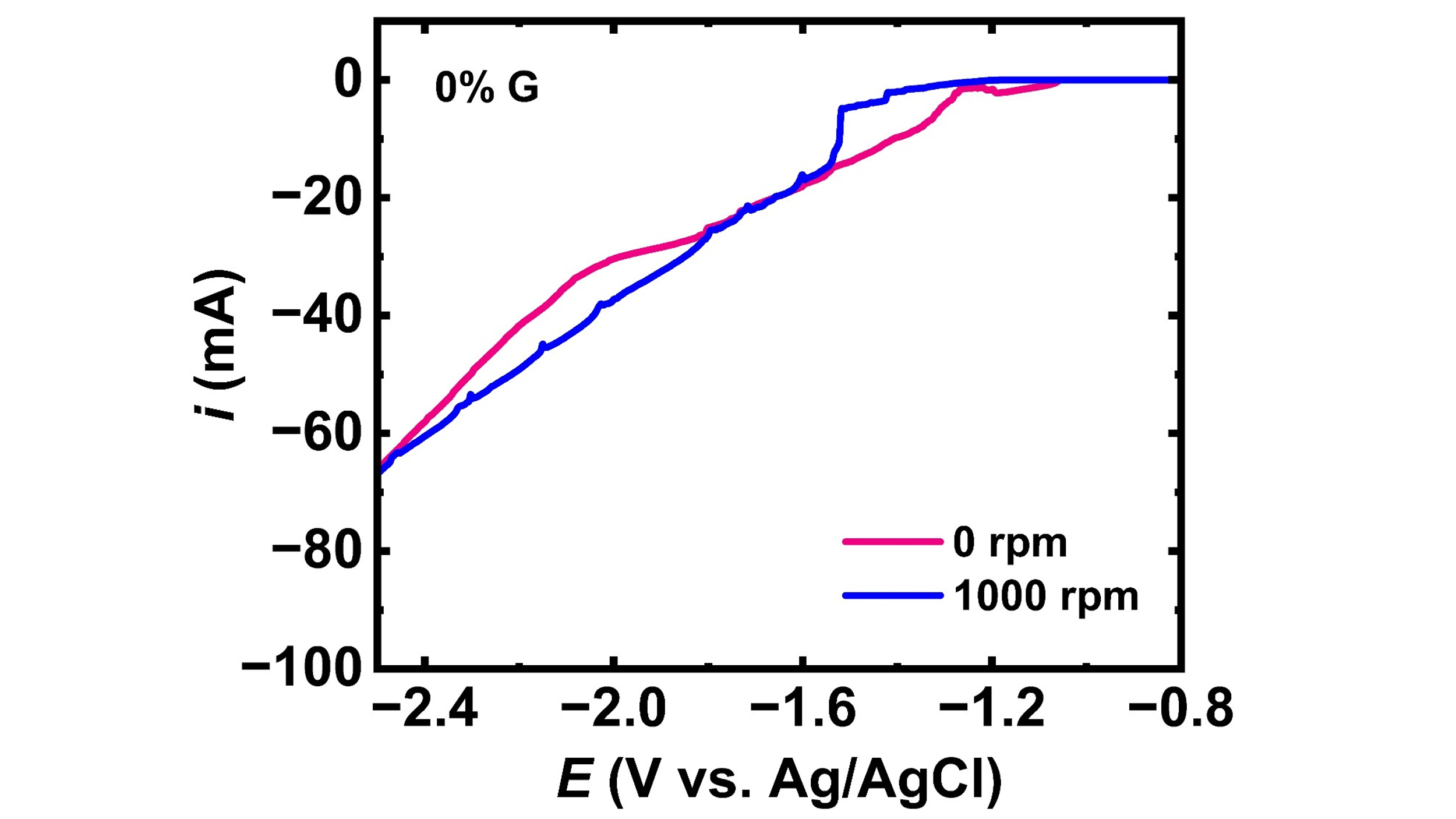


**Fig S15.** Linear sweep voltammograms of 1 M bare ZnCl_2_ solution (0% G) using the Rotating disk electrode setup.


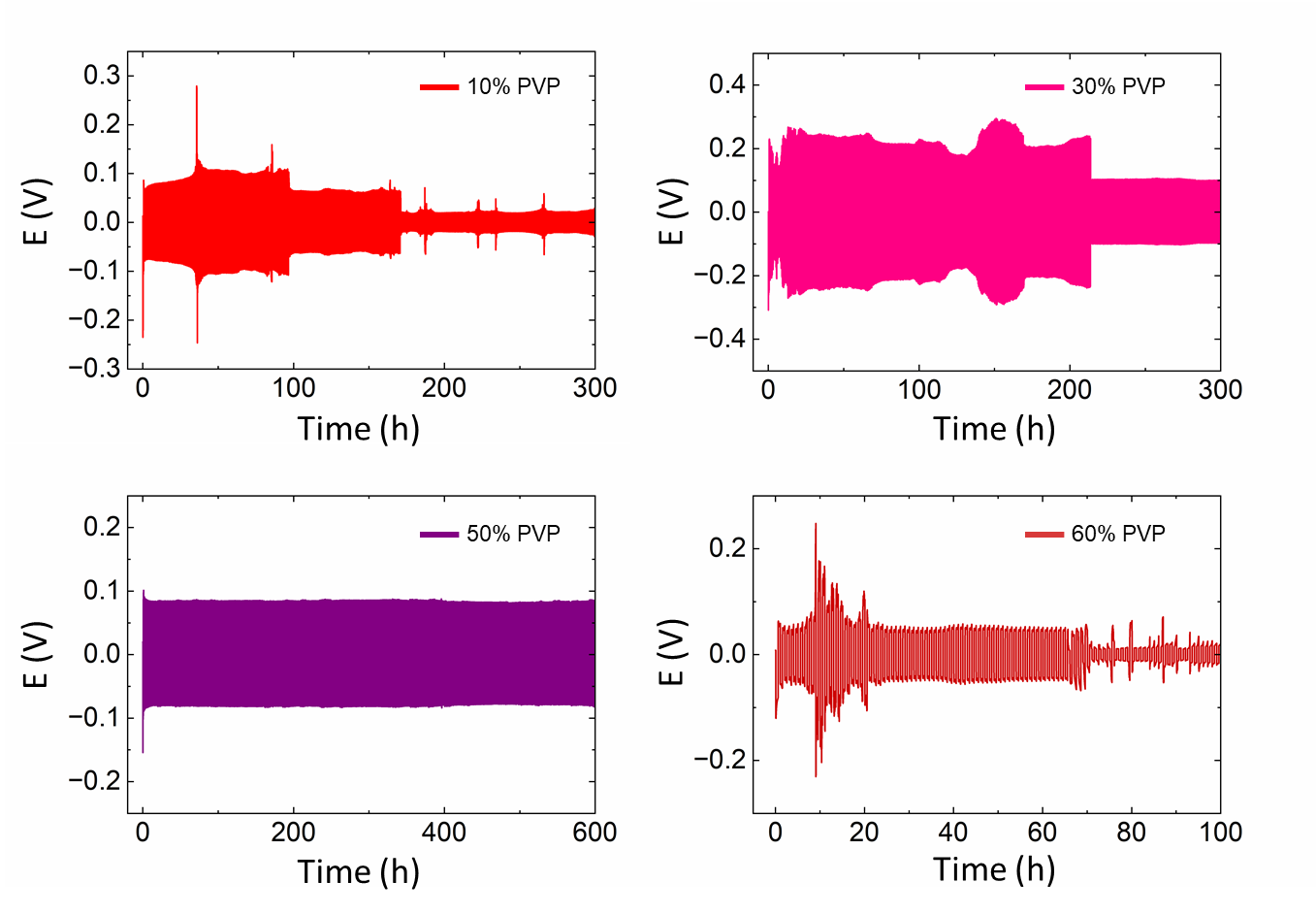


**Fig S16.** Zn|Zn symmetric cell cycling at a current density of 1 mA/cm² and a capacity of 0.5 mAh/cm² in 1 M ZnCl₂ under the varying concentrations of PVP.


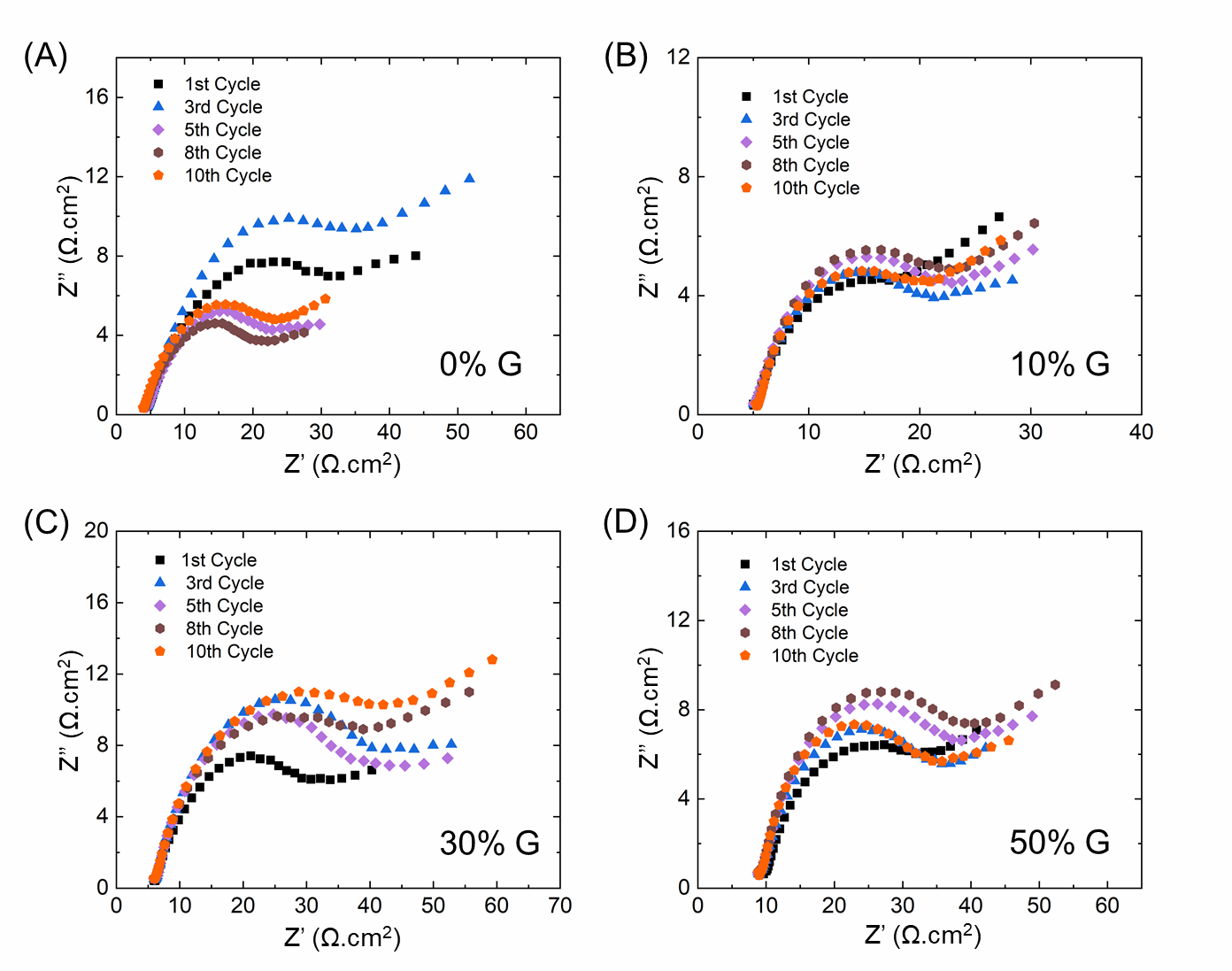


**Figure S17.** Electrochemical impedance spectroscopy (EIS) measured intermittently during cycling for electrolytes containing *(A)* 0% glycerol, *(B)* 10% glycerol (optimal G), *(C)* 30% glycerol, and *(D)* 50% glycerol. Nyquist plots were collected after the 1st, 3rd, 5th, 8th, and 10th cycles under identical conditions. The electrolyte with optimal glycerol concentration (10% G) exhibits the smallest variation in charge-transfer resistance and minimal evolution of the impedance response with cycling, whereas glycerol-free and high-glycerol electrolytes show pronounced R_ct_ growth and interfacial evolution, indicative of unstable zinc deposition and surface degradation.


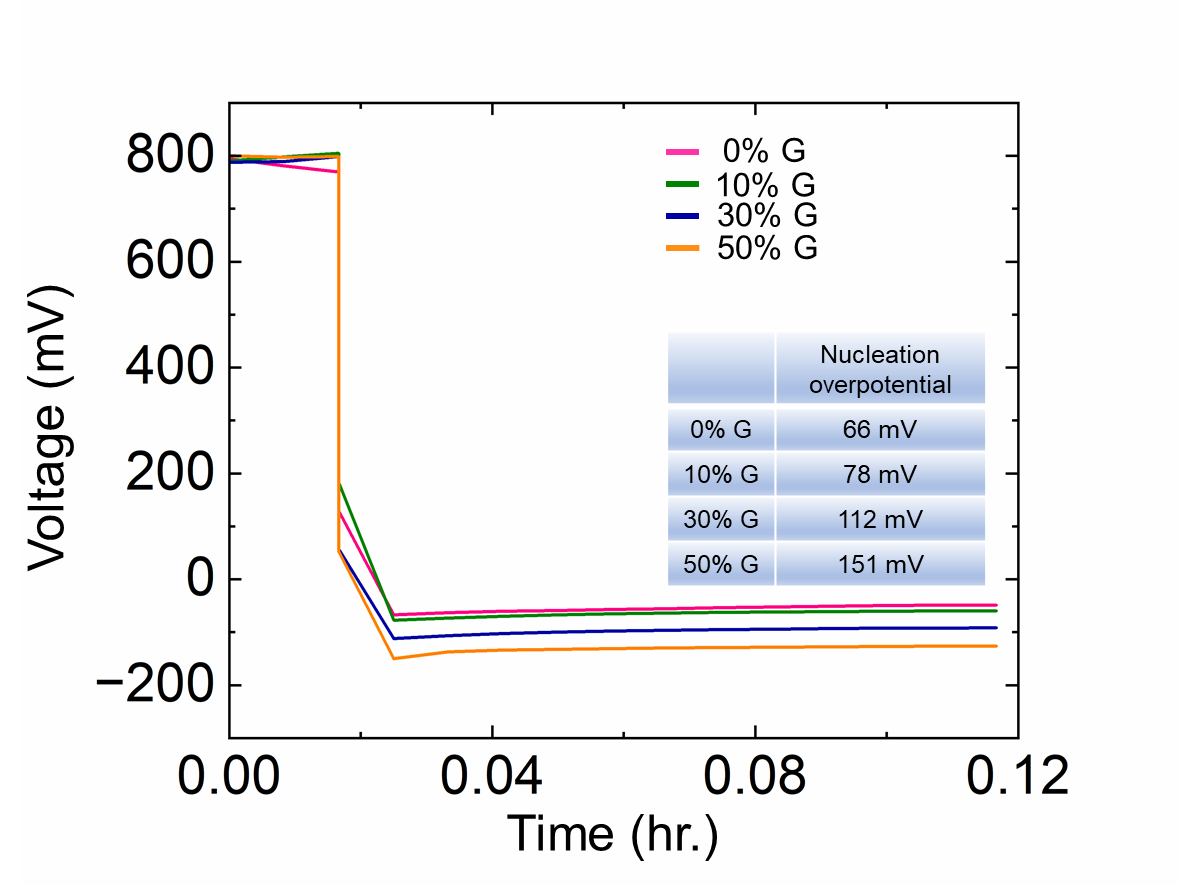


**Fig S18.** Voltage time profile of zinc electrodeposition on Cu foil showing increased nucleation overpotential with increased content of glycerol in 1 M ZnCl_2_.


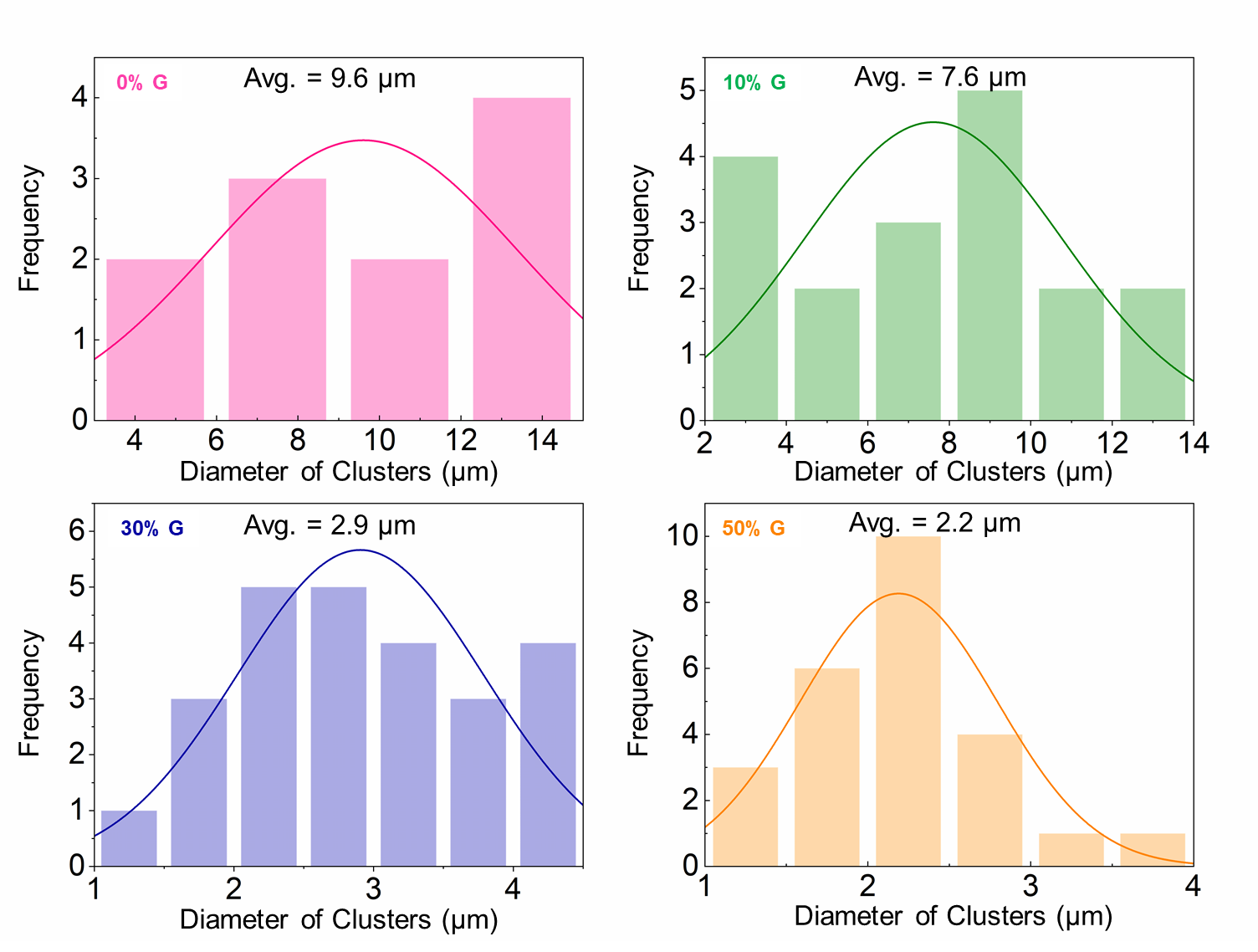


**Fig S19.** Cluster size histogram plot obtained from the SEM images showing smaller cluster size with increasing glycerol content in the electrolyte.


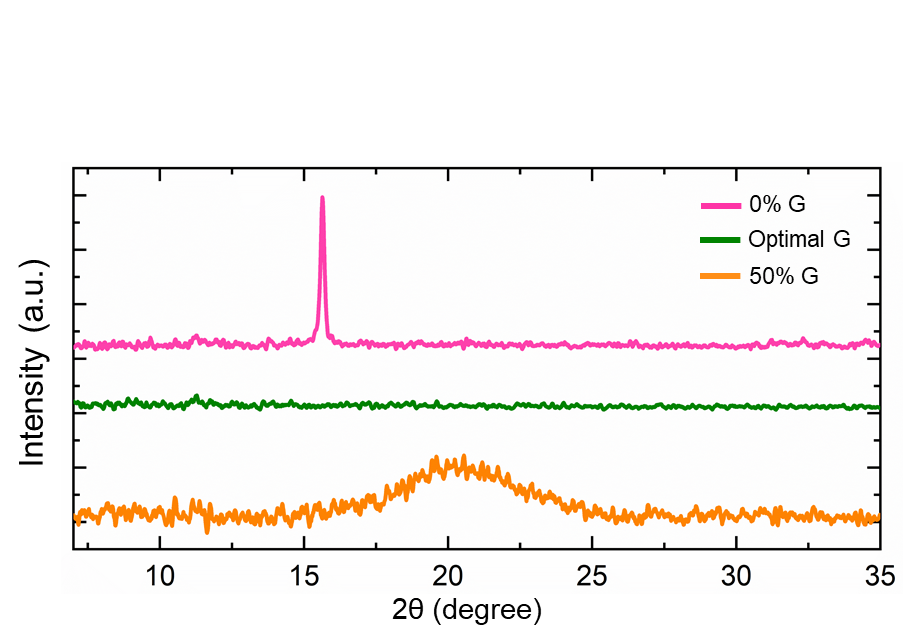


**Fig S20.** Low angle XRD patterns of electrodeposited Zn on Cu foil for different glycerol concentrations.


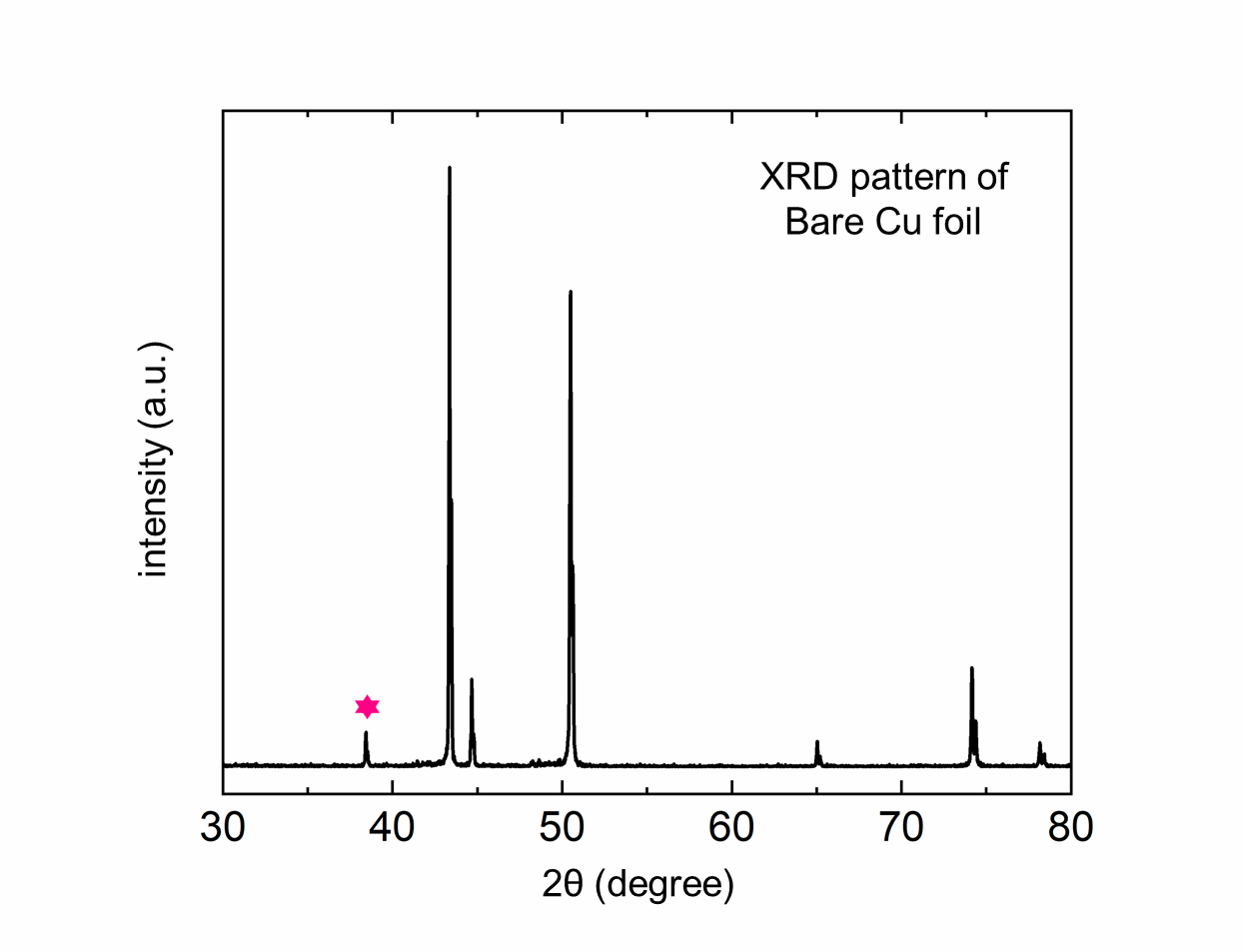


**Fig S21.** XRD pattern of bare Cu foil where the pink starred reflection is identical as seen in Fig. 6e


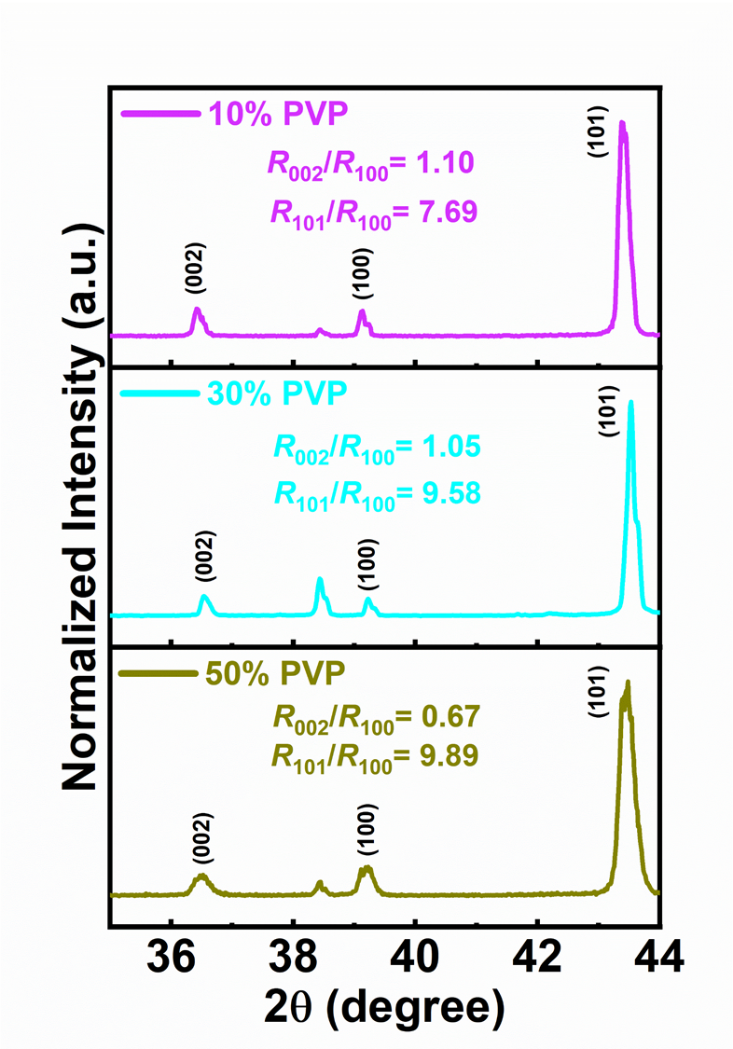


**Fig S22.** XRD patterns of electrodeposited Zn on Cu foil for different PVP concentrations.


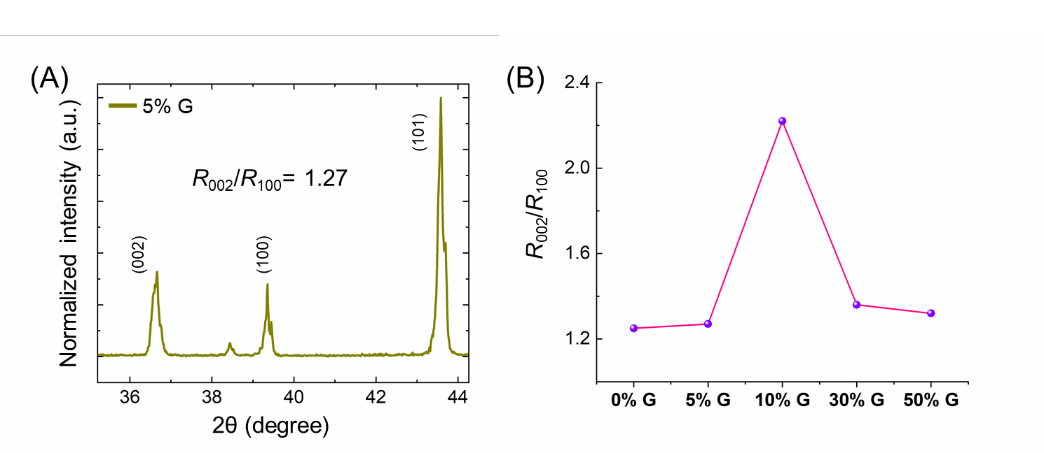


**Figure S23.** (a) X-ray diffraction (XRD) patterns of zinc deposits obtained from electrolytes containing 5% G. (b) Evolution of *R*_002_​/*R*_100_​​ as a function of glycerol concentration, showing that the 5% G data point follows the non-monotonic trend observed across the glycerol series. This behavior is consistent with coin-cell performance and supports the existence of an optimal kinetic window rather than a linear structure-composition relationship.


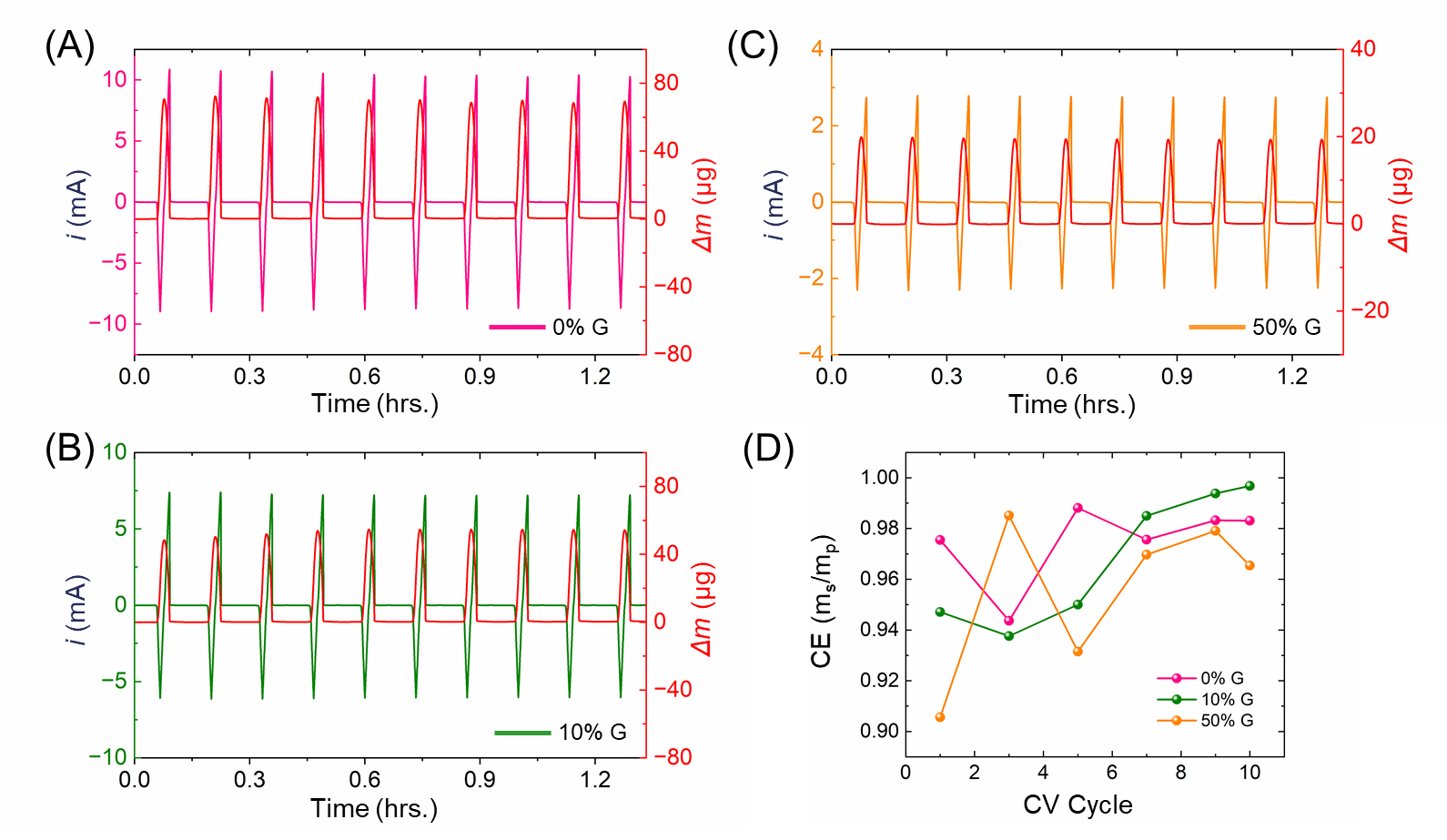


**Figure S24.** Electrochemical quartz crystal microbalance (EQCM) measurements of Zn deposition and stripping in electrolytes containing different glycerol concentrations (0%, 10%, and 50% G). (a-c) Measurements were performed over 10 consecutive cyclic voltammetry (CV) cycles under identical conditions. (d) The ratio of stripped mass to plated mass is shown to quantify mass reversibility during zinc cycling, where values closer to unity indicate minimized dead Zn formation and corrosion-derived irreversible mass accumulation.


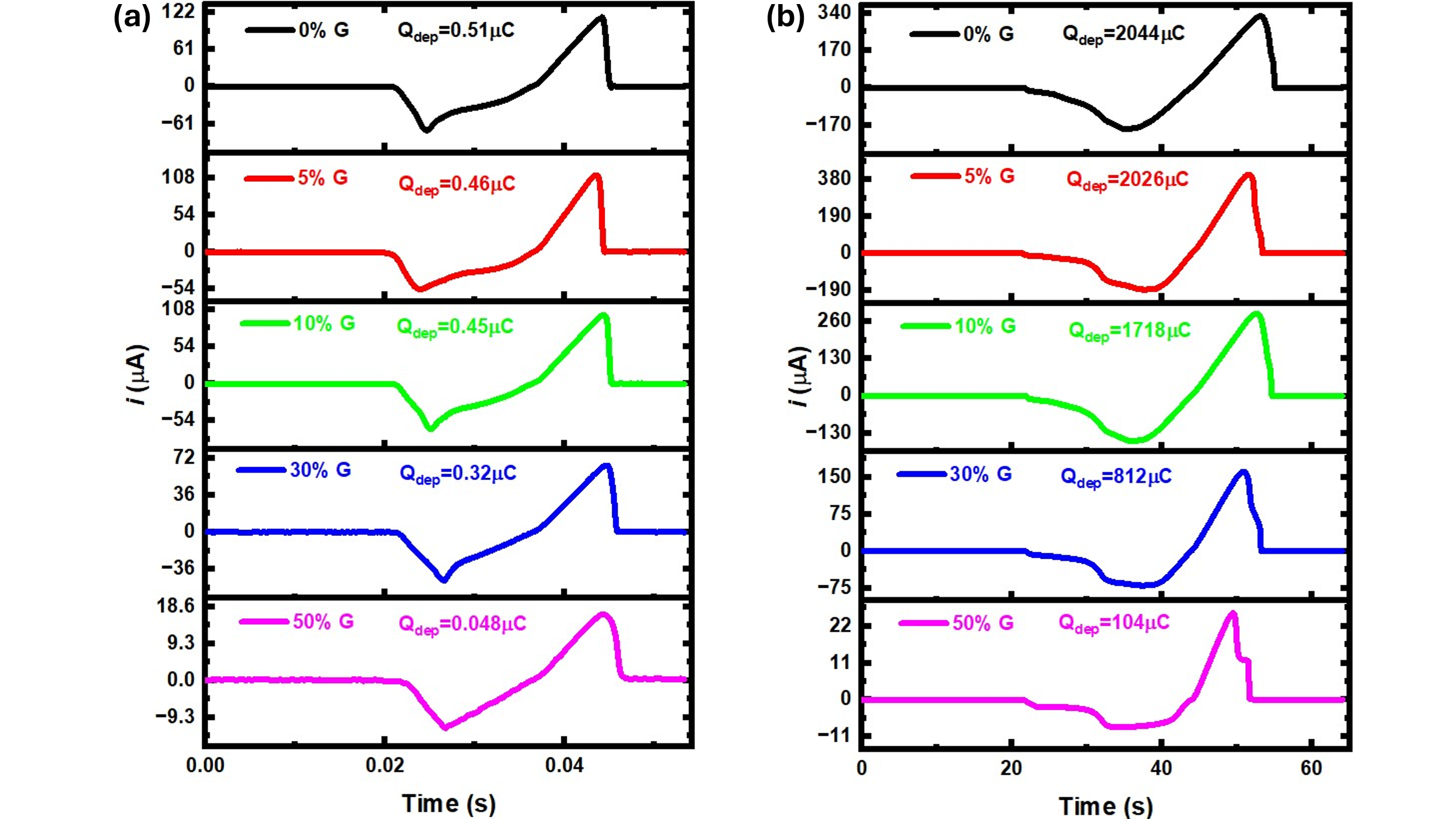


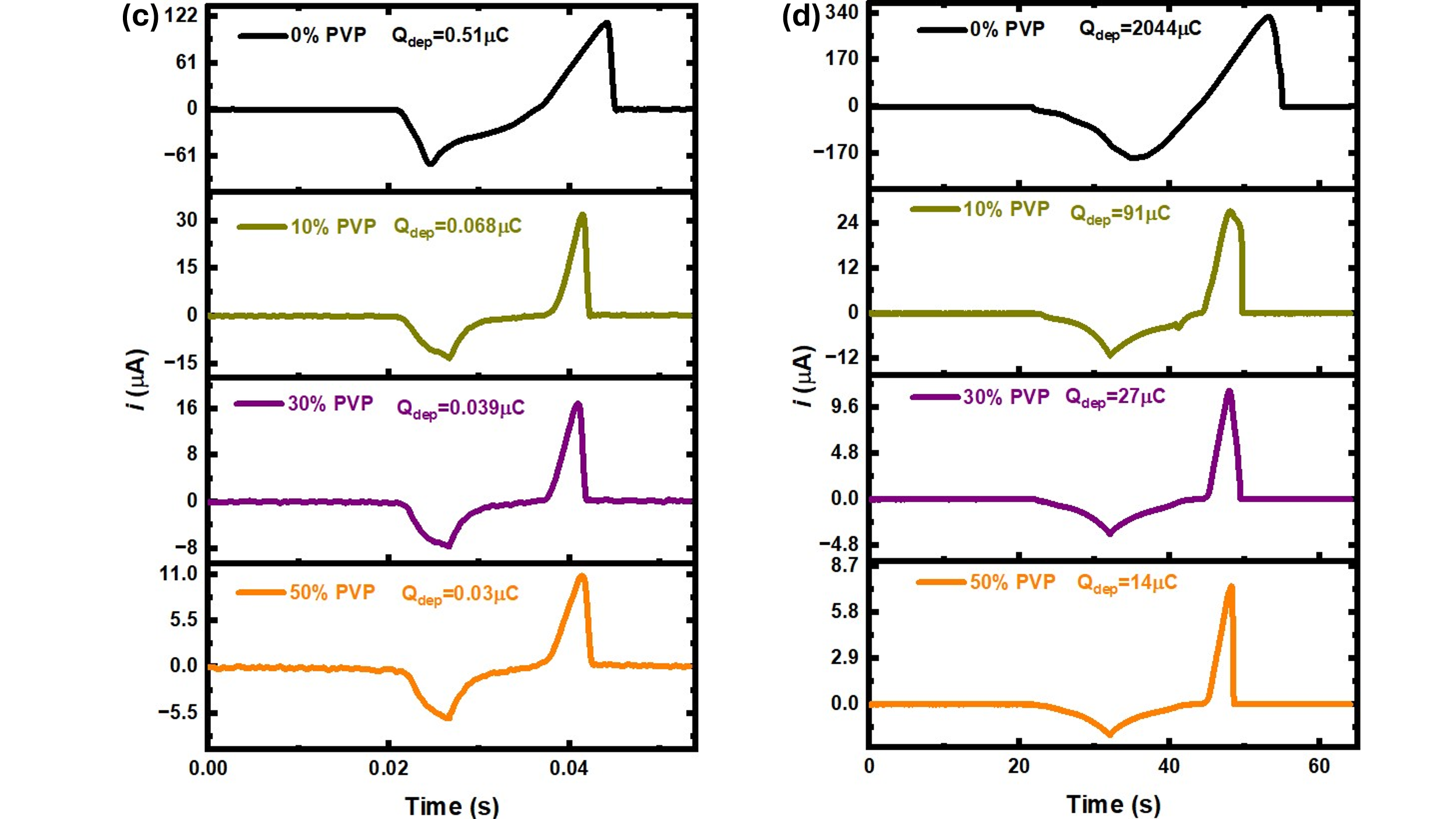


**Fig S25.** Current vs time plot of Zn electrodeposition for Glycerol on 12.5 µm rad. W ultramicroelectrode at scan rates (a) 0.05 Vs^-1^ and (b) 60 Vs^-1^ and for PVP at (c) 0.05 Vs^-1^ and (d) 60 Vs^-1^.

**
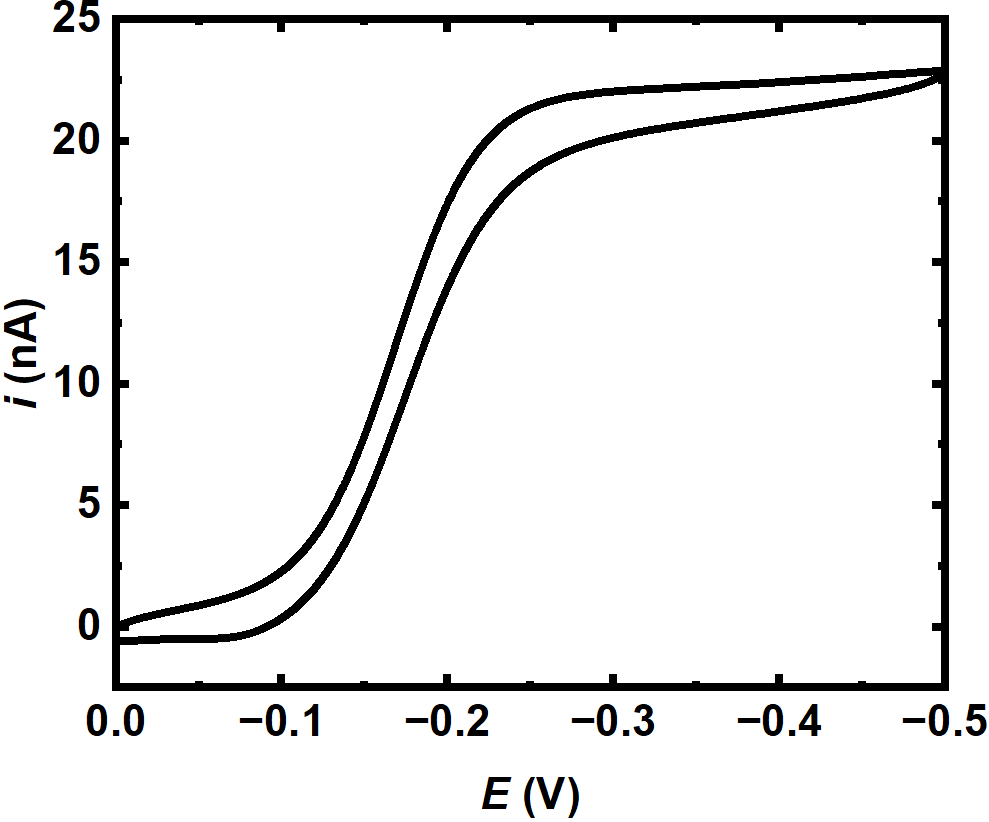
**

**Fig S26.** Cyclic voltammogram at 10 mV/s in 5 mM hexaammineruthenium(III) chloride using a two-electrode setup, with homemade W UME as the working electrode and Ag/AgCl in 1 M KCl serving as the counter/reference electrode.


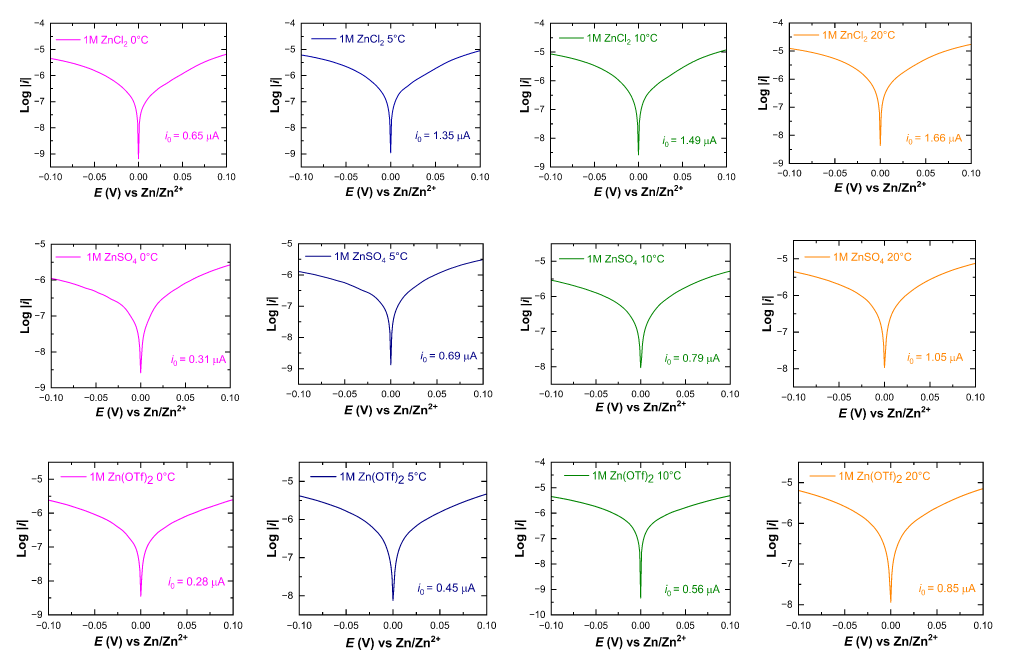


**Fig S27.** Tafel plots of 1 M ZnCl_2_, ZnSO_4_, and Zn(OTf)_2_ electrolytes extracted from the FSCVs at the scan rate of 30 Vs-1, in accordance with the methodology detailed in the main file. Here we have shown data for 0, 5, 10 and 20 °C for each of the electrolytes. Similarly three more sets of data have been collected from three independent FSCV experiments.

| **Electrolyte** | ***i*_0_ (μA)** | ***Q_D_*_ep_ (μC)** | **CE @ 60 V/s (%)** | **D_Zn2+_ (cm^2^/s)** |
| --- | --- | --- | --- | --- |
| ZnCl_2_ | 1.91 | 0.51 | 99.03 | 12×10^-6^ |
| 5% G | 1.63 | 0.46 | 99.10 | 11×10^-6^ |
| 10% G | 1.51 | 0.45 | 99.50 | 9×10^-6^ |
| 30% G | 0.84 | 0.32 | 98.70 | 5×10^-6^ |
| 50% G | 0.3 | 0.084 | 97.50 | 1×10^-6^ |
| 10% PVP | 0.49 | 0.068 | 98.95 | 6×10^-6^ |
| 30% PVP | 0.4 | 0.039 | 99.30 | 2×10^-6^ |
| 50% PVP | 0.38 | 0.03 | 99.70 | 1×10^-6^ |

**Table S1.** Exchange current ($i_{0}$​), deposited charge ($Q_{dep})$, and CE (%) as a function of concentration of Glycerol/PVP at 60 Vs^-1^.
